# Gene network and biological pathways associated with susceptibility to differentiated thyroid carcinoma

**DOI:** 10.1038/s41598-021-88253-0

**Published:** 2021-04-26

**Authors:** Om Kulkarni, Pierre-Emmanuel Sugier, Julie Guibon, Anne Boland-Augé, Christine Lonjou, Delphine Bacq-Daian, Robert Olaso, Carole Rubino, Vincent Souchard, Frédérique Rachedi, Juan Jesus Lence-Anta, Rosa Maria Ortiz, Constance Xhaard, Pierre Laurent-Puig, Claire Mulot, Anne-Valérie Guizard, Claire Schvartz, Marie-Christine Boutron-Ruault, Evgenia Ostroumova, Ausrele Kesminiene, Jean-François Deleuze, Pascal Guénel, Florent De Vathaire, Thérèse Truong, Fabienne Lesueur

**Affiliations:** 1 Inserm, U900, Institut Curie, PSL University, Mines ParisTech, 75248 Paris, France; 2 Université Paris-Saclay, UVSQ, Gustave Roussy, Inserm, CESP, 94807 Villejuif, France; 3 Université Paris-Saclay, CEA, Centre National de Recherche en Génomique Humaine, 91057 Evry, France; 4 Centre Hospitalier Territorial de Polynésie Française, CHTPF, Pirae, Tahiti, 98713 Papeete, French Polynesia; 5 Instituto Nacional de Oncologia y de Radiobiologia, INOR, La Havana, Cuba; 6 University of Lorraine, INSERM CIC 1433, Nancy CHRU, Inserm U1116, FCRIN, INI-CRCT, 54000 Nancy, France; 7 Centre de Recherche des Cordeliers, INSERM, Sorbonne Université, USPC, Université Paris Descartes, Université Paris Diderot, EPIGENETEC, 75006 Paris, France; 8 Registre Général des Tumeurs du Calvados, Centre François Baclesse, 14000 Caen, France; 9 Inserm U1086-UCNB, Cancers and Prevention, 14000 Caen, France; 10 Registre des Cancers Thyroïdiens, Institut Jean Godinot, 51100 Reims, France; 11 Environment and Radiation Section, International Agency for Research on Cancer, 69008 Lyon, France

**Keywords:** Cancer epidemiology, Cancer genomics, Thyroid cancer, Computational biology and bioinformatics, Risk factors

## Abstract

Variants identified in earlier genome-wide association studies (GWAS) on differentiated thyroid carcinoma (DTC) explain about 10% of the overall estimated genetic contribution and could not provide complete insights into biological mechanisms involved in DTC susceptibility. Integrating systems biology information from model organisms, genome-wide expression data from tumor and matched normal tissue and GWAS data could help identifying DTC-associated genes, and pathways or functional networks in which they are involved. We performed data mining of GWAS data of the EPITHYR consortium (1551 cases and 1957 controls) using various pathways and protein–protein interaction (PPI) annotation databases and gene expression data from The Cancer Genome Atlas. We identified eight DTC-associated genes at known loci 2q35 (*DIRC3*), 8p12 (*NRG1*), 9q22 (*FOXE1*, *TRMO*, *HEMGN*, *ANP32B*, *NANS*) and 14q13 (*MBIP*). Using the EW_dmGWAS approach we found that gene networks related to glycogenolysis, glycogen metabolism, insulin metabolism and signal transduction pathways associated with muscle contraction were overrepresented with association signals (false discovery rate adjusted p-value < 0.05). Additionally, suggestive association of 21 KEGG and 75 REACTOME pathways with DTC indicate a link between DTC susceptibility and functions related to metabolism of cholesterol, amino sugar and nucleotide sugar metabolism, steroid biosynthesis, and downregulation of ERBB2 signaling pathways. Together, our results provide novel insights into biological mechanisms contributing to DTC risk.

## Introduction

Differentiated thyroid carcinoma (DTC) is the most common type of endocrine cancer and accounts for 98% of all cases of thyroid cancer. It originates from epithelial follicular cells of the thyroid and includes three histological types, namely papillary thyroid carcinoma (PTC), follicular thyroid carcinoma (FTC), and Hürthle cell carcinoma^1^, with PTC representing about 85% of all thyroid malignancies^2^. DTC incidence varies considerably around the world with age-standardized incidence rates of 10.2 per 100,000 person-years in women and 3.1 per 100,000 person-years in men in 2018^3^. In most countries, DTC incidence has increased at a faster rate than most other malignancies during the last few decades. It is now the 5th most frequent cancer in women, whereas it was ranked 14th 20 years ago^3,4^. The causes underlying geographic, ethnic and temporal variations are still unknown. It could be explained by environmental and genetic factors, as well as changes in screening practices. In particular, some have attributed the increase in DTC incidence to improved diagnosis that leads to the detection of small tumors of minimal clinical relevance (microcarcinomas)^4^, whereas others argue that more sensitive diagnostic procedures cannot completely explain this increase of DTC rates^5^. The only well-established environmental risk factor for DTC is exposure to ionizing radiation during childhood and adolescence^6^ but it does not appear to have contributed importantly to these trends^7^. Anthropometric factors such as excess weight, tall height and large body size have also been consistently associated with risk of DTC^8–14^. In particular, a large meta-analysis showed that increase of weight, body mass index (BMI), waist or hip circumference and waist-to-hip ratio are associated with a greater risk of PTC, FTC and anaplastic thyroid cancer^15^. Because DTC occurs more frequently in women than in men, it was also suspected to be associated with hormonal and reproductive factors among women^7^. Thyroid cancer is also characterized by having one of the highest familial risk of any cancer supporting heritable predisposition^16^. In spite of such a high familial risk, few chromosomal loci have been implicated in DTC so far. Genome-wide association studies (GWAS)^17–23^ including ours^24^ identified mainly four DTC susceptibility loci at 9q22, 14q13, 2q35 and 8p12, which were replicated in different populations. However, the identified single nucleotide polymorphism (SNPs) were shown to account for only about 10% of the DTC familial risk, emphasizing that much remains to be discovered. Furthermore, all published studies examined genetic associations with DTC at the individual SNP or gene level. Data mining of GWAS data at a higher level of complexity using systems biology is still an under-explored topic. Of the seven GWAS performed for DTC, only one of the published datasets was additionally analyzed using pathway identification methods^21^. None of the studies employed protein–protein interaction (PPI) network-based methods to explore links between associated genes, and only two of them used expression quantitative trait loci (eQTL) data to identify potential causal regulatory sequence variants at DTC associated loci^21,23^. However, such approaches have been successful in identifying new susceptibility alleles for other complex traits. For instance, analysis of GWAS data using pathway-based enrichment methods successfully identified IL12/IL23 pathways associated with Crohn disease, involving genes that were subsequently identified as susceptibility genes only through meta-analysis of several GWAS^25^. Integrative analyses of GWAS data, eQTL and PPI networks also provided valuable biological insights in some complex diseases, such as Alzheimer disease^26^ and asthma^27^.

Here we re-analyzed the genome-wide genotyping data from seven case–control studies on DTC from the EPITHYR consortium using protein–protein interaction databases, various resources for pathway maps, as well as available eQTL data on DTC from The Cancer Genome Atlas (TCGA) to annotate SNPs and to identify biological mechanisms contributing to DTC susceptibility.

## Results

### Data set and results of the standard SNP-level analysis

We used GWAS data from the EPITHYR consortium^24^ that included subjects from case-control studies conducted in Metropolitan France (CATHY^11^, YOUNG-thyr^13^ and E3N^12^ studies), South Pacific Islands (Polynesia^9^ and New Caledonia^8^), Cuba^14^ and the Gomel region of Belarus, affected by the Chernobyl accident^28^. Characteristics of the study participants of European ancestry included in the analyses are described in Table 1.

**Table 1:** Characteristics of participants of the seven EPITHYR case–control studies used in the gene-, pathway- and network-level analyses.

Study	Cases		Controls	
	*N* = 1551	%	*N* = 1957	%
CATHY	450	29.0	533	27.2
Cuba	102	6.6	103	5.3
Chernobyl	66	4.3	304	15.5
E3N	276	17.8	287	14.7
New Caledonia	21	1.4	68	3.5
French Polynesia	0	0	4	0.2
YOUNG-Thyr	636	41.0	658	33.6
**Age (years)**				
[0–10]	5	0.3	43	2.2
[10–20]	115	7.4	301	15.4
[20–30]	378	24.4	425	21.7
[30–40]	335	21.6	382	19.5
[40–50]	199	12.8	239	12.2
≥ 50	519	33.5	567	29.0
Mean age [range]	40.6 [7–83]	–	37.0 [5–80]	–
**Sex**				
Female	1276	82.3	1508	77.1
Male	275	17.7	449	22.9
**Histology**				
Papillary	1414	91.2	–	–
Follicular	137	8.8	–	–

In the SNP-level analysis, 258 SNPs reached the standard genome-wide significance *P*-value threshold of 5 × 10^–8^. All SNPs were located in the known DTC susceptibility loci at 2q35, 8p12, 9q22.33 and 14q13.3 (Supplementary Figure 1A). No additional signal was evidenced when the analysis was restricted to PTC cases only (Supplementary Figure 1B).

### Gene-level analysis

According to GENCODE release 28, the analyzed SNPs were mapped to 19,120 protein-coding genes that were next used in the gene-based association test from VEGAS2^29^. This analysis identified eight genes associated with DTC with a false discovery rate adjusted p-value (*P*_FDR_) < 0.05, namely, *DIRC3*, *NRG1*, *FOXE1*, *TRMO*, *HEMGN*, *ANP32B*, *NANS* and *MBIP*, all of them being located at known DTC susceptibility loci (Table 2). The analysis restricted to PTC cases identified *TRIM14* at 9q22.33 in addition to these eight genes (Supplementary Table S1).

**Table 2:** Genes associated with DTC risk, SNPs and eQTL within or in the vicinity of these genes, and effect of eQTL on the expression of genes in cis.

Locus	Gene	Gene *P*_EMP_^a^	Gene *P*_FDR_^b^	#SNPs (*N*)^c^	Top SNP	OR_per allele_^d^	95%CI	*P*_per allele_	Cis-eQTL (*N*)^e^	eGene^f^
2q35	*DIRC3*	1.00 × 10^–7^	0.0038	451	rs16857611	1.42	1.28–1.58	1.25 × 10^–10^	223	*DIRC3*, *IGFBP5*
8p12	*NRG1*	2.00 × 10^–6^	0.0063	523	rs28406305	1.34	1.21–1.48	3.19 × 10^–8^	197	*NRG1*
9q22.33	*FOXE1*	1.00 × 10^–7^	0.0038	138	rs10739513	1.60	1.44–1.79	1.88 × 10^–17^	87	*TRMO*
9q22.33	*TRMO*	1.00 × 10^–7^	0.0038	92	rs7046645	1.58	1.42–1.77	9.85 × 10^–17^	64	*TRMO*
9q22.33	*HEMGN*	1.00 × 10^–7^	0.0038	61	rs7037324	1.49	1.35–1.65	8.03 × 10^–15^	31	*TRMO*, *NANS*
9q22.33	*ANP32B*	1.00 × 10^–7^	0.0038	39	rs56145417	1.30	1.18–1.43	1.91 × 10^–7^	4	*TRMO*, *NANS*
9q22.33	*NANS*	4.00 × 10^–6^	0.0095	20	rs7870926	1.30	1.18–1.43	2.08 × 10^–7^	2	*TRMO*, *NANS*
14q13.3	*MBIP*	3.00 × 10^–6^	0.0082	47	rs116909374	2.14	1.66–2.76	4.88 × 10^–9^	0	None

^a^Empirical p-value of the association test at the gene level.

^b^p-value of the association test with DTC risk at the gene level, after FDR correction.

^c^Number of analyzed SNP within the gene or at ± 50 kb from the gene boundaries.

^d^Per allele Odds Ratio (OR) for the top SNP at the gene locus.

^e^number of eQTL at the gene locus.

^f^Gene whose expression is affected by the cis-eQTL.

To get more insight in the genetic mechanisms of DTC, we interrogated whether SNPs in or nearby the associated genes were acting as cis-eQTLs (defined as a SNP within 1 Mb from the gene transcriptional start site) using transcriptome data from 497 DTC cases from TCGA available through the PancanQTL project^30^. We identified a number of cis-eQTL for *DIRC3*, *IGFBP5*, *NRG1*, *TRMO* and *NANS* (Table 2), indicating that SNPs at the associated loci could alter the regulation of the expression of these five genes.

### Pathway-level analysis

To clarify which biological pathways are involved in the etiology of DTC, we next used Vegas2Pathway which uses gene-based p-values from VEGAS2 and pathway definitions from Kyoto Encyclopedia of Genes and Genomes (KEGG)^31–33^, Reactome^34^ and Gene Ontology (GO)^35^ (Table 3). Out of 380 KEGG pathways, 361 were tagged by SNPs from our dataset. Of those, 21 pathways were associated with DTC risk with *P*_EMP_ < 0.05, with the top pathway being linked to cholesterol metabolism; however, none of the highlighted pathways were significant after correction for multiple testing (Supplementary Table S2). Only four of the 21 highlighted pathways involved one of the eight genes identified in the gene-level analysis, namely ‘Messenger RNA biogenesis’ (*ANP32B*), ‘Amino sugar and nucleotide sugar metabolism’ (*NANS*), ‘EGFR tyrosine kinase inhibitor resistance (*NRG1*) and ‘Transfer RNA biogenesis’ (*TRMO*) and the three latter pathways were not associated anymore with DTC after excluding SNPs tagging these candidate genes.

**Table 3:** Pathway definitions used in the pathway-level analysis.

Database	Definitions (*N*)	Definition tagged with oncoarray SNPs (*N*)	Definitions with *P*_EMP_^a^ < 0.05 (*N*)	Source	Version
KEGG (HSA and BRITE definitions)	380	361	21	https://www.genome.jp/kegg-bin/get_htext?hsa00001.keg	v88 (Oct, 2018)
REACTOME	2020	1698	75	https://reactome.org/download/current/ReactomePathways.txt	v66 (Sep 2018)
GO biological process	5214	5203	253	GO database: http://purl.obolibrary.org/obo/go/go-basic.obo GO annotations: https://ftp.ncbi.nlm.nih.gov/gene/DATA/gene2go.gz	(Oct 2018)
GO cellular component	655	652	38		
GO molecular function	1060	1059	31		

^a^Empirical p-value of the association test with DTC risk at the pathway level.

Out of 2020 Reactome pathways, 1698 included SNPs from our dataset. Of those, 75 definitions were associated with DTC risk with *P*_EMP_ < 0.05. After excluding SNPs tagging the eight candidate genes, the 16 pathways involving *NRG1* were not associated anymore with DTC (Supplementary Table S3). Gene Ontology (GO) definitions related to biological processes, molecular functions and cellular components were also investigated. Associated definitions with *P*_EMP_ < 0.05 are listed in (Supplementary Table S4).

To assess similarities between pathways associated with DTC at *P*_EMP_ < 0.05 identified with KEGG, Reactome and GO, we performed pairwise comparisons between definitions of the three databases. Pairs of pathways with Jaccard Index > 0.1 are shown in Supplementary Table S5. We found that 16 of the top KEGG pathways showed some similarity with some Reactome pathways, and 40, 4 and 6 KEGG pathways showed some similarity with GO biological processes, cell components and molecular functions, respectively, confirming the inter-feature dependencies of the pathways highlighted with the three pathway databases.

### Co-analysis of thyroid carcinoma gene expression and GWAS data

**Figure 1:**
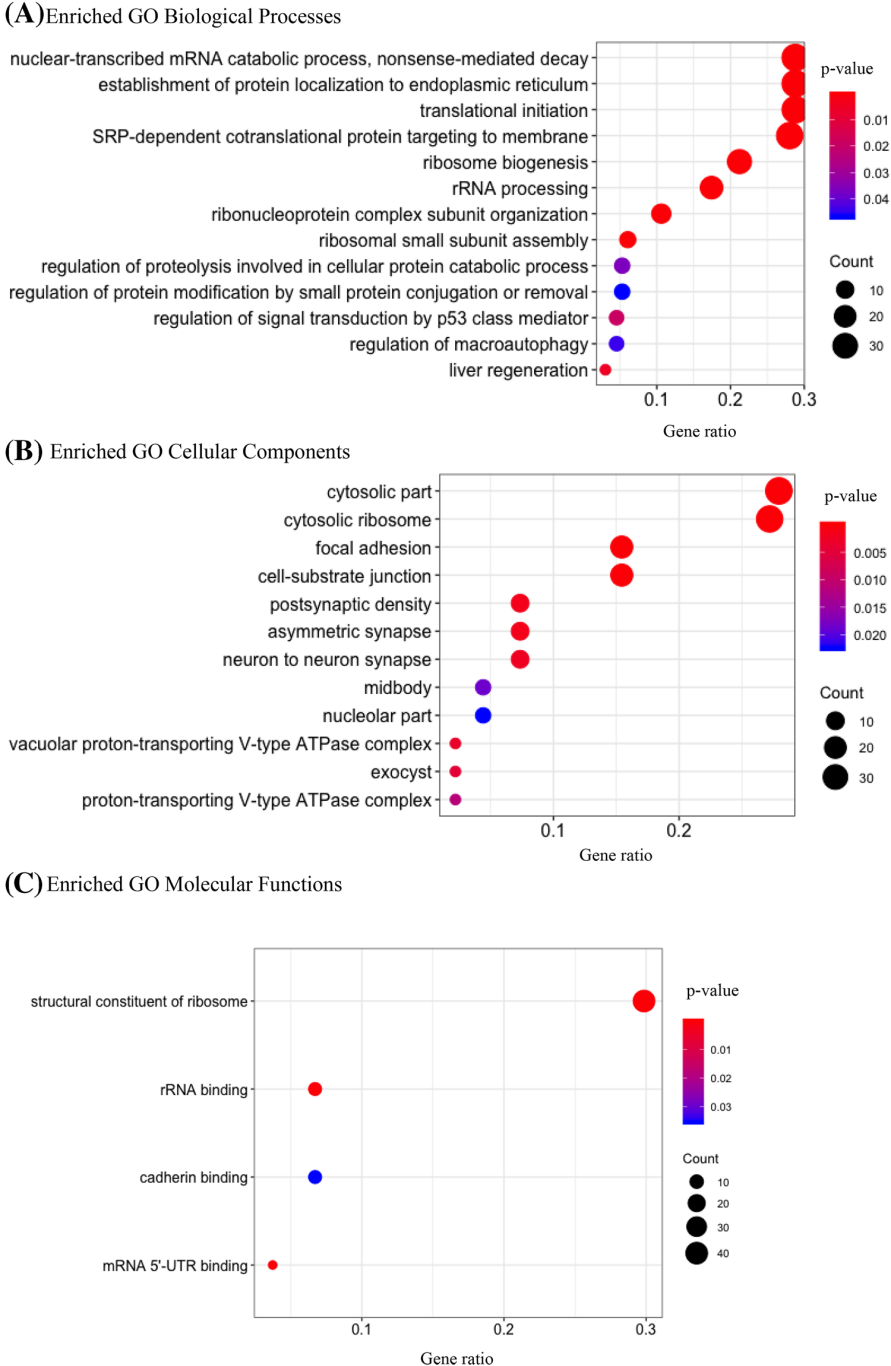
GO enrichment analysis using high throughput co-complex interaction annotations for (**A**) biological processes, (**B**) cellular components, (**C**) molecular functions. For each plot, Y-axis represents a significant GO definition, and X-axis represents the counts of enriched genes (Gene ratio). The gradient of color represents the different *p*-values, and size of the dot represents the count number of genes in each GO term.

To gain a deeper understanding of the genetic architecture of DTC, we then combined TCGA genomic expression data from 59 PTC/normal tissue sample pairs with PPI networks and EPITHYR GWAS data using the EW_dmGWAS approach^36^. The 19,129 genes containing OncoArray SNPs were involved in 4524 subnetworks describing binary interactions (that is direct PPI) and in 6590 subnetworks describing co-complex interactions. Among the 19,129 genes, 16,386 were differentially expressed between normal and tumor tissue. This information was used by the algorithm to assign edge weights to the nodes of the subnetworks to rank them for downstream gene enrichment analysis. Hence, the top 1% subnetworks contributing to DTC susceptibility involved 72 genes with binary interactions and 143 genes with in co-complex interactions. Using Reactome pathway definitions, we found that five pathways were significantly enriched, including ‘Glycogen breakdown (glycogenolysis)’ (*P*_FDR_ = 7.9 × 10^–3^), ‘Glycogen metabolism’ (*P*_FDR_ = 2.5 × 10^–2^) and two pathways related to muscle contraction when binary interactions annotations were considered (Table 4). Furthermore, we found 47 Reactome pathways significantly enriched when co-complex interactions annotations were considered (Table 4). Using GO definitions, we found 14 biological processes, 12 cellular components and 4 molecular functions associated with DTC (Fig. 1) while with KEGG definitions, only the ‘Ribosome’ (*P*_FDR_ = 2.7 × 10^–43^) and ‘starch and sucrose metabolism’ (*P*_FDR_ = 4.6 × 10^–2^) pathways were significantly enriched.

**Table 4:** Reactome pathways enriched with genes involved in the top 1% subnetworks obtained when considering binary and co-complex interactions.

Interactions type	Enriched reactome pathway	Genes (*N*)	*P*_EMP_^a^	*P*_FDR_^b^
Binary	Striated muscle contraction	7	2.2 × 10^–10^	7.3 × 10^–08^
	Muscle contraction	9	3.6 × 10^–07^	5.9 × 10^–05^
	Glycogen breakdown (glycogenolysis)	3	7.3 × 10^–05^	7.9 × 10^–03^
	Glycogen metabolism	3	3.2 × 10^–04^	2.5 × 10^–02^
	The role of GTSE1 in G2/M progression after G2 checkpoint	4	3.9 × 10^–04^	2.5 × 10^–02^
Co-complex	Peptide chain elongation	37	4.4 × 10^–53^	1.2 × 10^–50^
	Viral mRNA translation	37	4.4 × 10^–53^	1.2 × 10^–50^
	Formation of a pool of free 40S subunits	38	1.6 × 10^–52^	3.0 × 10^–50^
	Eukaryotic translation elongation	37	3.4 × 10^–52^	3.1 × 10^–50^
	Selenocysteine synthesis	37	3.4 × 10^–52^	3.1 × 10^–50^
	Eukaryotic translation termination	37	3.4 × 10^–52^	3.1 × 10^–50^
	Nonsense mediated decay (NMD) independent of the exon junction complex (EJC)	37	9.1 × 10^–52^	7.2 × 10^–50^
	L13a-mediated translational silencing of ceruloplasmin expression	38	1.3 × 10^–50^	8.7 × 10^–49^
	GTP hydrolysis and joining of the 60S ribosomal subunit	38	1.9 × 10^–50^	1.2 × 10^–48^
	Nonsense-mediated decay (NMD)	38	6.3 × 10^–50^	3.1 × 10^–48^
	Nonsense mediated decay (NMD) enhanced by the exon junction complex (EJC)	38	6.3 × 10^–50^	3.1 × 10^–48^
	Eukaryotic translation initiation	38	2.9 × 10^–49^	1.2 × 10^–47^
	Cap-dependent translation initiation	38	2.9 × 10^–49^	1.2 × 10^–47^
	SRP-dependent cotranslational protein targeting to membrane	37	1.5 × 10^–48^	5.9 × 10^–47^
	Selenoamino acid metabolism	37	1.5 × 10^–47^	5.3 × 10^–46^
	Influenza viral RNA transcription and replication	38	4.0 × 10^–47^	1.4 × 10^–45^
	Major pathway of rRNA processing in the nucleolus and cytosol	41	5.8 × 10^–47^	1.9 × 10^–44^
	Regulation of expression of SLITs and ROBOs	40	8.3 × 10^–46^	2.5 × 10^–44^
	Influenza life cycle	38	9.3 × 10^–46^	2.7 × 10^–44^
	rRNA processing in the nucleus and cytosol	41	6.2 × 10^–45^	1.7 × 10^–43^
	Influenza Infection	38	2.2 × 10^–44^	5.8 × 10^–43^
	rRNA processing	41	5.8 × 10^–44^	1.5 × 10^–42^
	Signaling by ROBO receptors	40	3.2 × 10^–41^	7.8 × 10^–40^
	Translation	40	5.9 × 10^–36^	1.6 × 10^–34^
	Infectious disease	41	1.9 × 10^–32^	4.2 × 10^–31^
	Metabolism of amino acids and derivatives	39	1.9 × 10^–30^	4.1 × 10^–29^
	Formation of the ternary complex, and subsequently, the 43S complex	19	5.1 × 10^–26^	1.0 × 10^–24^
	Translation initiation complex formation	19	9.4 × 10^–25^	1.8 × 10^–23^
	Ribosomal scanning and start codon recognition	19	9.4 × 10^–25^	1.8 × 10^–23^
	Activation of the mRNA upon binding of the cap-binding complex and eIFs, and subsequent binding to 43S	19	1.4 × 10^–24^	2.5 × 10^–23^
	TCR signaling	6	1.1 × 10^–3^	2.0 × 10^–2^
	Regulation of mRNA stability by proteins that bind AU-rich elements	5	1.7 × 10^–3^	3.0 × 10^–2^
	FBXL7 down-regulates AURKA during mitotic entry and in early mitosis	4	1.9 × 10^–3^	3.2 × 10^–2^
	Insulin receptor recycling	3	2.1 × 10^–3^	3.3 × 10^–2^
	Regulation of RUNX3 expression and activity	4	2.1 × 10^–3^	3.3 × 10^–2^
	Insulin processing	3	2.3 × 10^–3^	3.5 × 10^–2^
	Stabilization of p53	4	2.4 × 10^–3^	3.5 × 10^–2^
	Iron uptake and transport	4	2.5 × 10^–3^	3.7 × 10^–2^
	Downstream TCR signaling	5	2.8 × 10^–3^	3.9 × 10^–2^
	G2/M transition	7	3.0 × 10^–3^	4.2 × 10^–2^
	Mitotic G2-G2/M phases	7	3.2 × 10^–3^	4.2 × 10^–2^
	rRNA modification in the nucleus and cytosol	4	3.2 × 10^–3^	4.2 × 10^–2^
	Transferrin endocytosis and recycling	3	3.4 × 10^–3^	4.4 × 10^–2^
	Cilium assembly	7	3.5 × 10^–3^	4.4 × 10^–2^
	ROS, RNS production in phagocytes	3	3.8 × 10^–3^	4.6 × 10^–2^
	p53-dependent G1 DNA damage response	4	4.0 × 10^–3^	4.7 × 10^–2^
	p53-dependent G1/S DNA damage checkpoint	4	4.0 × 10^–3^	4.7 × 10^–2^

^a^Empirical p-value of the association test with DTC risk at the pathway level.

^b^p-value of the association test with DTC risk at the pathway level, after FDR correction.

## Discussion

Incorporating gene network and pathway classification tools in GWAS data analysis can point toward significantly overrepresented molecular pathways, which had not been picked up in traditional single-SNP analysis due to the stringent genome-wide significance level and to the limited power of some case–control studies to identify low-risk alleles. To our knowledge, this is the first study on DTC susceptibility where integrative analyses of GWAS data, gene expression data in tumor, and biological pathways or physical PPI network data were performed to gain biological insights in the disease. Data mining of the EPITHYR GWAS data using several systems biology annotation tools and various analysis strategies has allowed to identify high confidence candidate pathways for subsequent analyses to be further explored to understand the underlying mechanisms of DTC carcinogenesis. Indeed, although the EPITHYR GWAS is one of the GWAS with the largest number of DTC cases reported so far (1551 cases and 1957 controls of European ancestry), new findings from the classical per-SNP analysis were limited and the eight candidate genes (*DIRC3, NRG1, FOXE1, TRMO, HEMGN, ANP32B, NANS* and *MBIP*) identified in the gene-level analysis were all located in the well characterized DTC susceptibility loci 2q35, 8p12, 9q22.33, and 14q13.3^22^. Moreover, a functional link between these candidate genes could not clearly be established at this point.

SNPs in the nuclear long noncoding RNA *DIRC3* (*disrupted in renal cancer 3*) have been associated with both thyroid stimulating hormone level and DTC risk^19^, and it was shown that *DIRC3,* playing a role in tumor invasion and multifocality, represents a potential prognostic factor for PTC^37^. Interestingly, the top SNP for *DIRC3*, rs16857611, is an eQTL which downregulates the expression of *DIRC3* and the expression of its neighboring tumor suppressor gene *IGFBP5* whose product belongs to a family of proteins which interacts with insulin-like growth factors (IGFs) involved in regulation of vital processes such as cell proliferation, differentiation and apoptosis. In melanoma, it was shown that DIRC3 activates expression of *IGFBP5* through modulating chromatin structure and suppressing SOX10 binding to putative regulatory elements^38^, suggesting that the two genes at the 2q35 could represent potential therapeutic targets for both melanoma and DTC.

*NRG1* encodes the membrane glycoprotein Neuregulin 1, which acts on the erb-b2 receptor tyrosine kinase (ERBB) family of tyrosine kinase receptors. It is the major HER3 ligand, which promotes its engagement with HER2 kinase and the subsequent transphosphorylation of HER3. It is involved in regulation of *MAPK* and *AKT* signaling pathways which are involved in thyroid carcinoma cells proliferation and survival^39^. *FOXE1*, is a thyroid-specific transcription factor essential for thyroid gland development and maintenance of the differentiated state. In vitro studies in thyroid cancer cell lines revealed that FOXE1 modulates cell migration, suggesting a role in epithelial-to-mesenchymal transition^40^. *HEMGN*, also known as *EDAG-1* (*Embryonic develop-associated gene 1*) is upregulated in thyroid carcinoma tissues and cells, and it has been proposed to regulates the proliferation and apoptosis of cells via PI3K/Akt signaling pathway^41^.

*ANP32B* (*Acidic Nuclear Phosphoprotein 32 Family Member B)* is a multifunctional protein working as a cell cycle progression factor as well as an anti-apoptotic protein is involved in hepatocellular carcinoma^42^. The gene product of *MBIP* regulates the JNK pathway which is involved in intracellular signaling of thyroid and other human cancers^43^. A role for *TRMO* encoding a tRNA methyltransferase involved in tRNA processing, and for *NANS* involved in sialic acid synthesis process in tumorigenesis has not been evidenced so far although variation in the expression of the two genes has been observed in thyroid carcinoma according to TCGA transcriptomic data, suggesting that further studies on these candidates should be pursued.

Network and pathway tools were developed for computational gene prioritization to make use of functional information from gene and protein databases to gain more insights in disease-related biological mechanisms. They are, therefore, biased toward the well-studied genes; interactions and pathways and SNPs in non-coding genes (lncRNA, miRNA, and snRNA) and in intergenic regions are omitted. Here, we assigned SNPs that lie within 50 kb on either side of a gene’s coding sequence boundaries to compute its association *p* value which is used by pathways and networks centric approaches. With this gene definition, only 1951 (0.4%) OncoArray SNPs that passed QC were not linked to a gene.

Our study also illustrates that the alternative representation of the same biological pathway (*e.g.* in KEGG, Reactome and GO) may influence the results of the statistical enrichment analysis and that pathway-centric approaches employed to interpret -omics data rely on the choice of the pathway databases used. This is because pathways are often described at varying level of detail, with diverse data types and with vaguely defined boundaries. In particular, KEGG includes pathway maps such as for metabolism, genetic, and environmental information processing, while Reactome is based on biological reactions (binding, activation, translocation, degradation) and GO is a hierarchy of terms representing biological processes, molecular functions and cellular components. We chose to use these three databases that differ in the average number of pathways they contain, the average number of proteins per pathway, the types of biochemical interactions they incorporate, and the subcategories that they provide (e.g. signal transduction, genetic interaction, and metabolic) to gain a comprehensive overview of pathway landscapes altered in DTC. Reassuringly, we found that, although limited in number, similar pathways named differently across databases were associated with DTC with comparable p-values.

We also found that the EW_dmGWAS approach combining association, differential gene co-expression profile and functional interaction analyses was more informative than the standard pathway-based approaches to prioritize gene sets. The integrative analysis showed that genes involved in ‘muscle contraction’, ‘glycogen’ and ‘insulin’ related pathways play a role in the etiology of DTC. Using this approach, KEGG definitions, GO biological processes and GO molecular functions were also significantly enriched for ribosome-related pathways, and GO cellular components were enriched for several nervous system related terms.

Although top ranked pathways highlighted in the standard pathway analyses with VEGAS2 did not achieve statistical significance, some are in line with those evidenced with EW_dmGWAS or play a role in the development of other carcinomas, and therefore could help prioritizing the best candidates for therapeutic intervention. For instance, VEGAS2 analyses suggested involvement of cholesterol homeostasis pathways in DTC and indicate that MAPK pathway, involved in melanoma and other cancer types^44,45^, and steroid biosynthesis related pathways, involved in prostate cancer^45^ could also been altered in DTC. Other top ranked pathways related to NCAM1, a neural cell adhesion molecule shown to be involved in development of the nervous system as well as in cancer metastasis^46^ or to ERBB2 and other growth factors acting in thyroid tumorigenesis were also evidenced.

Gene-networks and pathways highlighted in this study were identified using the European subset of EPITHYR only, due to the limited sample size and heterogeneity in the population structure in other ethnic groups. Since allele frequencies of SNPs and DTC risk associated to them may vary from one population to another, pathway- or network-guided GWAS analysis in larger non-European samples will be useful to confirm the association with biological functions identified in Europeans and also to identify new ones. The major advantage of approaches such as EW_dmGWAS or similar approaches like the weighted gene co-expression network analysis (WGCNA)^47,48^ is their capability to perform biologically relevant dimension reduction as a result of the analysis. However, they use results of transcriptomic data analysis which reflect the inherent complexity of multiple biological processes. Moreover, data generated from different platforms also lead to noise and error generated by variations in experiment also affect the accuracy to distinct different samples. Further improvement of the algorithms is therefore needed to facilitate identification of causal hub genes involved in molecular mechanisms that could be used as therapeutic targets of the disease. Building methods using multitype data such as gene expression data, transcriptomic data and protein data will help to identify more accurate and reliable pathways as biological markers of disease. Alternatively, deep learning models may be used to jointly learn features from different type of omics data and then predict the key genes forming the modules, as such multi-task methods have been proposed for image classification in other complex diseases^49^.

To summarize, the strongest associations were found for gene sets acting in insulin resistance, amino sugar and nucleotide sugar metabolism-related pathways, which trigger weight gain, overweight or obesity reported to be positively associated with in thyroid cancer risk^50^. In EPITHYR, data on weight and height are available for all participants, and association between anthropometric factors and DTC risk was investigated separately in all studies^8–10,12–14^. In all studies, weight, height and BMI were positively associated with DTC risk. High body surface area was also investigated in three of the studies^10,13,14^, and it was also found to increase DTC risk. These results support the relevance of the above-mentioned pathways in DTC susceptibility. Genes sets acting in signaling pathways involved in muscle contraction, were also evidence in the EW-dmGWAS analysis. Interestingly, a recent GO term and KEGG pathway enrichment analysis performed on mRNA microarray datasets for human thyroid carcinomas and adenomas indicated that some biological functions of genes that were differentially expressed in the tumors included protein binding, cardiac muscle cell potential involved in contraction^51^, indicating that these functions play a role in both thyroid cancer development and progression. Hence, translating EPITHYR GWAS data into biologically relevant pathways and gene sets expands our knowledge on the potential mechanisms underlying DTC carcinogenesis, and provides evidence for the future development of clinically relevant of multigenic predictors for identifying individuals at high risk. Further population, clinical and laboratory research is needed to confirm our findings. Strategies to accelerate functional biological follow-up may include replication of the findings in other populations, fine-mapping, experimental studies such as metabolomic analyses to fully understand the biology and functional nature of the loci involved in signal transduction pathways associated with muscle contraction, glycogenolysis, and insulin metabolism in DTC susceptibility.

## Materials and methods

### Study participants

Study participants consisted of DTC cases and cancer-free controls of European descent originated from metropolitan France, New Caledonia, French Polynesia, Cuba and Gomel region of Belarus contaminated after the Chernobyl fallout, and who had been enrolled in one of the seven case–control studies from the EPITHYR consortium, with available blood or saliva DNA sample. After quality controls of the genotyping data (see next paragraph), 1551 cases and 1957 controls were used for all further analyses (Table 1). The study designs have been described in detail previously^8,11–13,52–54^. All studies provided information on histology of the tumor, ethnicity, personal and familial history of thyroid disease, menstrual and reproductive factors, exogenous hormone use, weight, height, dietary habits and residential and occupational histories. DTC cases with missing histology and individuals related at the first, second and third degree according to their genotypic data (i.e. 19 individuals, data not shown) were excluded.

Participants from all studies provided written informed consent. The present study was performed in compliance with the Helsinki Declaration and to the reference methodology from the National Committees for personal data protection in medical research.

CATHY, YOUNG-Thyr, E3N and New Caledonian studies were approved by the French ethics committee “Comité de Protection des Personnes” and the French data protection authority “Commission Nationale de L’informatique et des Libertés” (CNIL). The French Polynesian study was approved by the Ethical committee of French Polynesia and the CNIL. The Cuban study was approved by the Clinical Research Ethics Committee of the National Institute of Oncology, Havana, Cuba. The Chernobyl study was approved by the International Agency for Research on Cancer ethics committee and the Belarus Coordinating Council for Studies of the Medical Consequences of the Chernobyl Accident.

### Genotyping data

All individuals were genotyped at the Centre National de Recherche en Génomique Humaine (CNRGH/CEA) with the Infinium OncoArray beadchip (Illumina) designed to target over 530,000 SNPs across the genome^55^. For the purpose of EPITHYR studies, the beadchip was augmented with 13,759 SNPs known or suspected to be involved in DTC susceptibility or in thyroid hormone metabolism^24^. Standard genotyping array QC steps were applied to filter out SNPs which were either duplicate SNPs (814 SNPs) pseudo autosomal SNPs (37 SNPs), monomorphic SNPs (5210 SNPs) or SNPs deviating from Hardy Weinberg Equilibrium (HWE), *i.e.* applying HWE p-value thresholds of 10^–7^ for controls and 10^–12^ for cases, as performed by the OncoArray consortium in other studies^55^ (563 SNPs). In addition, SNPs with call rate per study < 95% (8327 SNPs) or showing cluster plot discordancy (4083 SNPs) were also discarded. This left 460,437 SNPs, of which 458,486 were located within or at ± 50 kb of a protein coding gene. In total, 3508 individuals with European descent (1551 cases and 1957 controls) as identified using ancestry markers and standard procedures described by the OncoArray consortium^55^ were used for all further analyses.

### SNP-level analysis

SNPs were tested individually with the assumption of an additive genetic model, using an unconditional logistic regression model adjusted for age (age at diagnosis for cases and age at inclusion for controls), sex, study and the first ten principal components to correct for population stratification. Analyses were performed with PLINK software v1.9^56^.

### Gene-level analysis

Gene-level analyses were performed using VEGAS2v02^29^. As we described in another work, VEGAS2 “performs gene-based tests based on association test from single variant analyses and accounts for linkage disequilibrium (LD) between SNPs and number of SNPs tested to avoid an increase in false positive results due to genes with multiple, highly correlated markers”^57^, Following the same strategy as what we reported previously, “we considered a SNP to belong to a gene if located within 50 kb on either side of the gene’s transcribed region, which we found to be a good balance between incorporating short-range regulatory variants while maintaining the specificity of the result for a specific gene, as variants associated with neighboring genes can influence the test statistic for a gene of interest”^57^. All SNPs were provided to the tool which assigns SNPs to genes and calculates gene-based empirical association p-values. The results shown were obtained using EPITHYR European controls as reference dataset for LD calculation. For SNP annotation, the latest GENCODE28 definitions mapped to hg19 were downloaded (ftp://ftp.ebi.ac.uk/pub/databases/gencode/Gencode_human/release_28/GRCh37_mapping/gencode.v28lift37.annotation.gff3.gz). Only protein coding definitions (*N* = 20,298) were used for the gene-based association tests.

Multiple testing was taken into account by using the Benjamini and Hochberg’s procedure to compute the FDR, with a statistical significance threshold of 0.05. The same gene level analysis was repeated for after excluding 137 FTC cases, using all the same parameters.

### Annotation of eQTLs

We used the PancanQTL database^30^ (bioinfo.life.hust.edu.cn/PancanQTL/) to search for eQTL within or nearby genes associated with DTC risk in EPITHYR. This database provides access to eQTL-based analysis of genotype and expression data of 9196 tumor samples in 33 cancer types obtained from TCGA. For the present study, we downloaded the cis-eQTL identified in the 497 DTC samples analyzed in the PancanQTL project (https://portal.gdc.cancer.gov/).

### Pathway-level analysis

Pathway-level analysis were performed using Vegas2Pathway^58^, which accounts for LD between the tested markers, and corrects for gene and pathway sizes. This test uses the VEGAS2 output and external pathway definitions. Here we used the reference biological pathway annotation databases KEGG, considering the HSA and BRITE hierarchies^31–33^, GO^35^ and Reactome^34^. The latest definitions for each database were downloaded. Number of definitions and number of OncoArray SNPs tagging genes involved in these definitions are provided in Table 3. The GO terms were subjected to filtering on basis of pathway size by only considering definitions with number of genes between 10 and 400. In addition, to further reduce the number of overlapping GO term definitions, a similarity measure (Jaccard index^59^) was calculated for each pair of GO terms. Two terms were considered "highly similar" if their Jaccard index was > 0.85, in that case only the largest set was kept. Internally, VEGAS2Pathway only considers pathway definitions having a minimum of five genes. The statistical test used by VEGAS2Pathway is similar to the test used by VEGAS2 but considering a gene set as a pathway definition. For each pathway-based test, an FDR correction with a statistical significance threshold of 0.05 was applied to correct for multiple testing.

### Gene network analysis

We used the EW_dmGWAS algorithm to investigate joined association signals beyond single markers^36^. EW_dmGWAS first annotates sets of genes using PPI networks as described in the HINT database (HINTDB), which collates interactions from BioGRID, MINT, iRefWeb, DIP, IntActa, HPRD, MIPS and the PDB^60^. Interactions are defined as either: *binary*, that is a direct biophysical interaction between two proteins, or *co-complex* associations, which means co-membership in a group, without implying direct pairwise interaction. These definitions of interactions, also called “co-complex categories” are divided into either literature-curated or deduced from high throughput experiments. Literature curated definitions include interaction data from thousands of small-scale studies focused at validating a single or a few specific hypotheses, while high throughput experiments produce large-scale interaction maps. Here we considered the high throughput definitions, for both binary (*N* = 47,427) and co-complex (*N* = 102,807) sets of interactions. EW_dmGWAS also uses data on condition-specific differential gene co-expression profiles to assign edge weights to the nodes of the PPI networks to prioritize gene sets (also called modules or subnetworks) for downstream gene enrichment analysis. Here we were interested in prioritizing genes that are differentially expressed in thyroid tumor tissue versus adjacent normal tissue samples. We used expression data from TCGA and selected tumor/normal tissue sample pairs for PTC cases available through the TCGA firebrowse portal (http://firebrowse.org/)^61^. Entire dataset in the file “illuminahiseq_rnaseqv2-RSEM_genes_normalized” was downloaded, and the TCGA barcodes were used to find matching tumor and healthy tissue by parsing the ‘sample’ field (https://docs.gdc.cancer.gov/Encyclopedia/pages/TCGA_Barcode/) The sample field has values with range 01–09 for tumor types, and range 10–19 for normal types, using this criteria we found 59 matching tumor and normal sample pairs. In brief, EW_dmGWAS integrates GWAS signals and gene expression profiles to extract subnetworks from a background PPI network. Node weights are derived from GWAS signals and edge weights are derived from gene expression profiles. Modules are ranked according to their score which is a combination of node weight and edge weight.

EW_dmGWAS was executed for each set of binary and co-complex interactions listed in HINTDB using gene-level association test p-values. For each category of interactions, only the top 1% modules were considered for use in gene enrichment analysis. Reactome gene enrichment analysis was performed with the R package ReactomePA^62^ and KEGG and GO gene enrichment analyses were performed with R package clusterProfiler^63^. Specifically, the functions *enrichPathway*, *enrichKEGG* and *enrichGO* were used. For GO annotations, a preprocessing step was necessary using the *simplify* function from clusterProfiler in order to remove redundant GO terms in the enrichment analysis.

### Disclaimer

Where authors are identified as personnel of the International Agency for Research on Cancer/World Health Organization, the authors alone are responsible for the views expressed in this article and they do not necessarily represent the decisions, policy or views of the International Agency for Research on Cancer/World Health Organization.

## Supplementary Information

Supplementary Information.
