# Supplementary material for: Gene network and biological pathways associated with susceptibility to differentiated thyroid carcinoma
Source: Sci Rep. 2021 Apr 26;11:8932. doi: 10.1038/s41598-021-88253-0 (PMC8076215; doi:10.1038/s41598-021-88253-0)
Supplement: Supplementary file 1 — Supplementary Information. [file 41598_2021_88253_MOESM1_ESM.docx]

**Gene networks and biological pathways associated with susceptibility to differentiated thyroid carcinoma**

Om Kulkarni^1^, Pierre-Emmanuel Sugier^2^, Julie Guibon^1,2^, Anne Boland-Augé^3^, Christine Lonjou^1^, Delphine Bacq-Daian^3^, Robert Olaso^3^, Carole Rubino^2^, Vincent Souchard^2^, Frédérique Rachedi^4^, Juan Jesus Lence-Anta^5^, Rosa Maria Ortiz^5^, Constance Xhaard^2,6^, Pierre Laurent-Puig^7^, Claire Mulot^7^, Anne-Valérie Guizard^8,9^, Claire Schvartz^10^, Marie-Christine Boutron-Ruault^2^, Evgenia Ostroumova^11^, Ausrele Kesminiene^11^, Jean-François Deleuze^3^, Pascal Guénel^2^, Florent De Vathaire^2^, Thérèse Truong^2^*, Fabienne Lesueur^1^*^¶^

*Equal contribution.

1. Inserm, U900, Institut Curie, PSL University, Mines ParisTech, F-75248 Paris, France
2. Université Paris-Saclay, UVSQ, Gustave Roussy, Inserm, CESP, F-94807, Villejuif, France
3. Université Paris-Saclay, CEA, Centre National de Recherche en Génomique Humaine, F-91057, Evry, France
4. Centre Hospitalier Territorial de Polynésie Française, CHTPF, Pirae, F-98713, Papeete, Tahiti, Polynésie Française
5. Instituto Nacional de Oncologia y de Radiobiologia, INOR, La Havana, Cuba
6. University of Lorraine, INSERM CIC 1433, Nancy CHRU, Inserm U1116, FCRIN, INI-CRCT, F-54000, Nancy, France
7. Centre de Recherche des Cordeliers, INSERM, Sorbonne Université, USPC, Université Paris Descartes, Université Paris Diderot, EPIGENETEC, F-75006, Paris, France
8. Registre Général des Tumeurs du Calvados, Centre François Baclesse, F-14000, Caen, France
9. Inserm U1086 -UCNB, Cancers and Prevention, F-14000, Caen, France
10. Registre des Cancers Thyroïdiens, Institut Jean Godinot, F-51100 Reims, France
11. Environment and Radiation Section, International Agency for Research on Cancer, F-69008, Lyon, France

**Supplementary Figure 1.** Manhattan plot showing result of the genome-wide association study for the EPITHYR European sample. (A) Results from 1551 DTC cases and 1957 controls. (B) Results when restricting the analysis to 1414 PTC cases and 1957 controls. Red line indicates threshold for genome-wide significance, *i.e*. *P*$\leq$5 x 10^-8^. Blue line indicates threshold for suggestive association, *i.e.* *P*$\leq$5 x 10^-6^. Numbers in red and blue indicate number of SNPs with p-value below the corresponding thresholds at 2q35, 8p12, 9q22, 14q13.

(A)


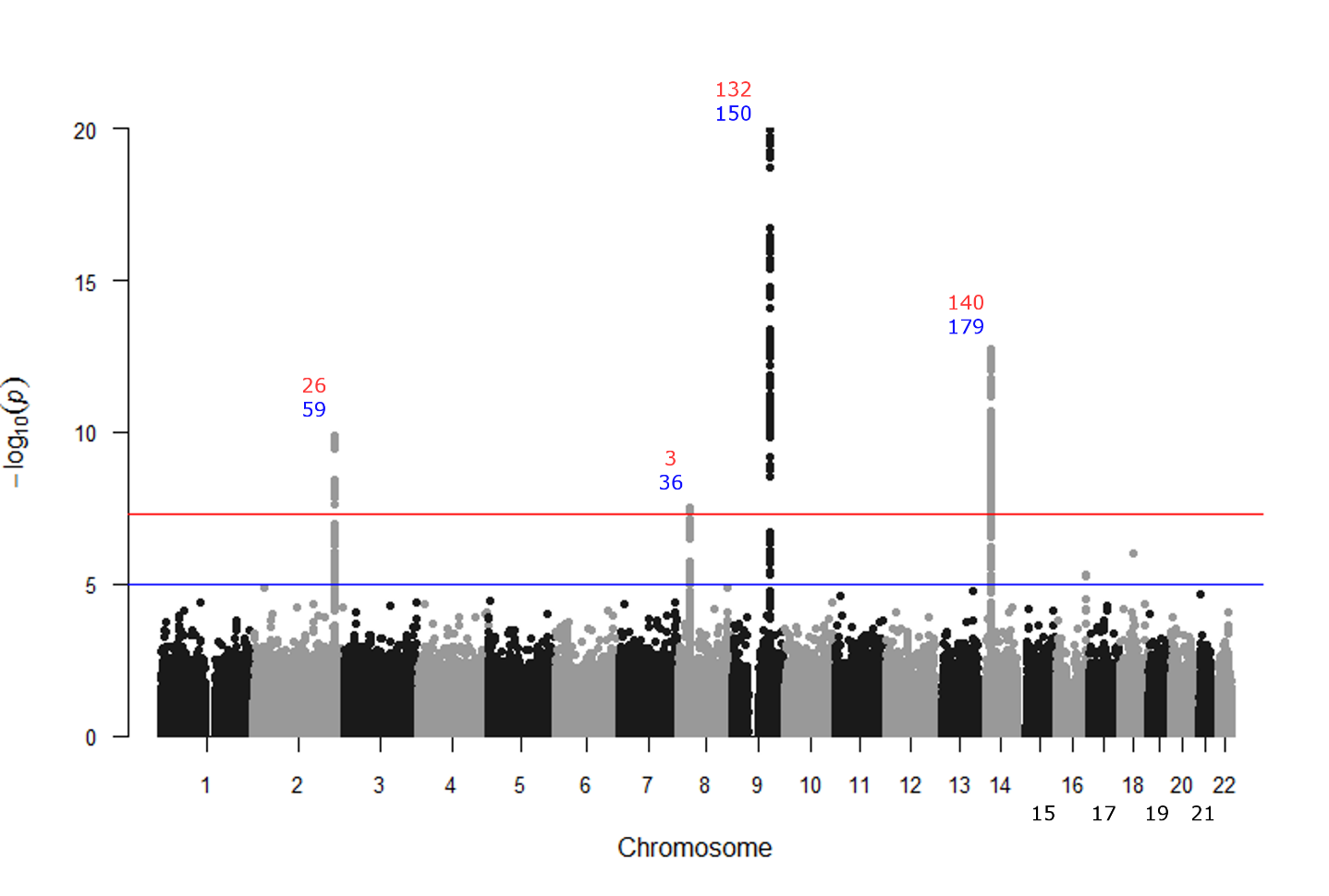


(B)


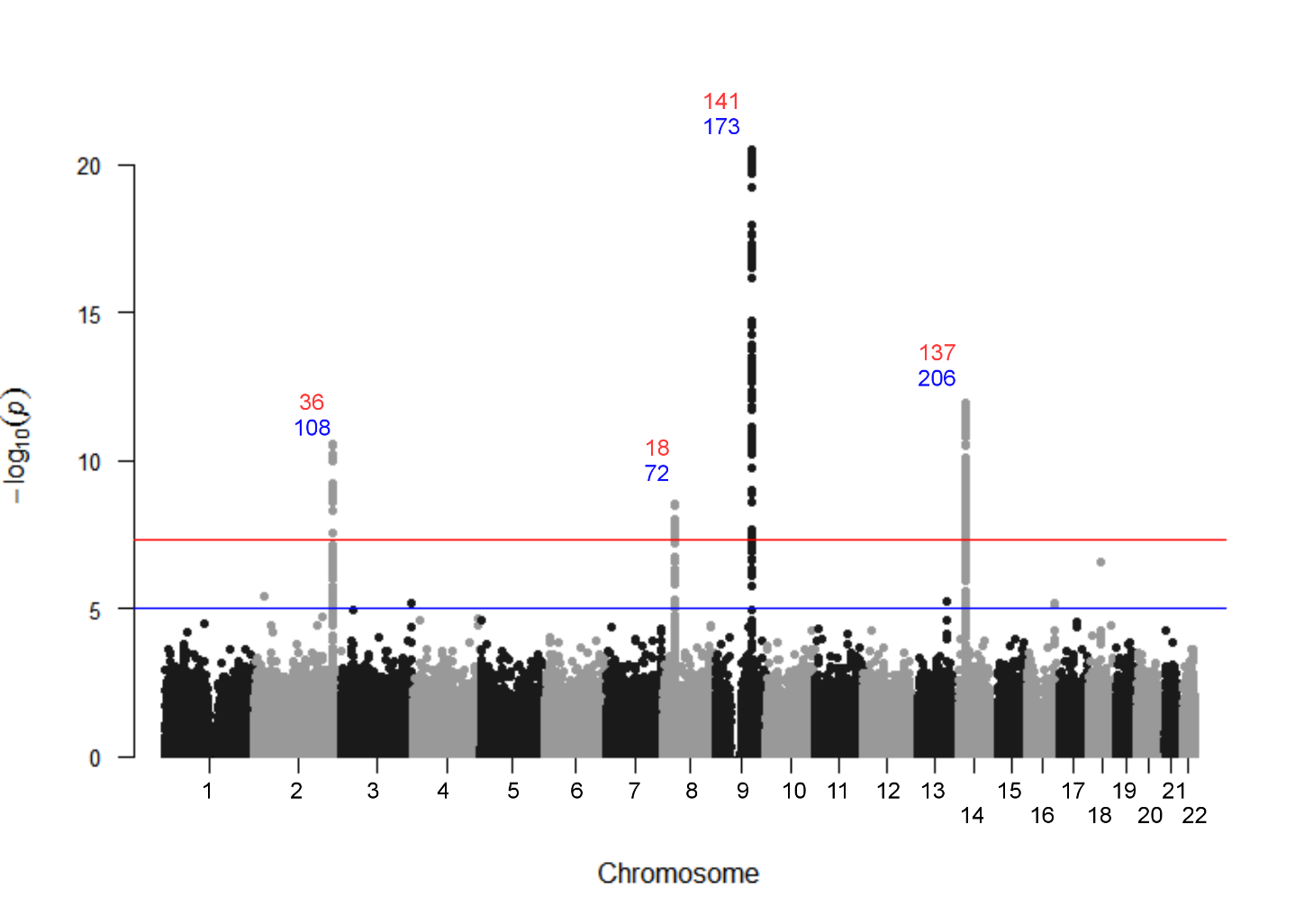


**Supplementary Table S1.** Genes associated with DTC risk when restricting the analysis to PTC cases.

| **Locus** | **Gene** | **Gene** | **Gene** | **#SNPs (*N*)^c^** | **Top SNP** | **OR_per allele_^d^** | **95%CI** | ***P*_per allele_** |
| --- | --- | --- | --- | --- | --- | --- | --- | --- |
|  |  | ***P*_EMP_^a^** | ***P*_FDR_^b^** |  |  |  |  |  |
| 2q35 | *DIRC3* | 1.00 x 10^-7^ | 0.0032 | 451 | rs16857611 | 1.46 | 1.30-1.63 | 2.83 x 10^-11^ |
| 8p12 | *NRG1* | 2.00 x 10^-6^ | 0.0047 | 523 | rs28406305 | 1.37 | 1.24-1.52 | 3.03 x 10^-9^ |
| 9q22.33 | *FOXE1* | 1.00 x 10^-7^ | 0.0032 | 138 | rs10739513 | 1.65 | 1.48-1.84 | 1.17 x 10^-18^ |
| 9q22.33 | *TRMO* | 1.00 x 10^-7^ | 0.0032 | 92 | rs7046645 | 1.69 | 1.46-1.81 | 5.93 x 10^-18^ |
| 9q22.33 | *HEMGN* | 1.00 x 10^-7^ | 0.0032 | 61 | rs7037324 | 1.51 | 1.36-1.68 | 5.79 x 10^-15^ |
| 9q22.33 | *ANP32B* | 1.00 x 10^-7^ | 0.0032 | 39 | rs56145417 | 1.33 | 1.20-1.47 | 2.11 x 10^-8^ |
| 9q22.33 | *NANS* | 2.00 x 10^-6^ | 0.0047 | 20 | rs7870926 | 1.33 | 1.20-1.47 | 2.30 x 10^-8^ |
| 9q22.33 | *TRIM14* | 1.40 x 10^-5^ | 0.0290 | 28 | rs7870926 | 1.33 | 1.20-1.47 | 2.30 x 10^-8^ |
| 14q13.3 | *MBIP* | 1.00 x 10^-7^ | 0.0032 | 46 | rs116909374 | 2.16 | 1.66-2.79 | 5.88 x 10^-9^ |

^a^ Empirical p-value of the association test at the gene level.

^b^ p-value of the association test with DTC risk at the gene level, after FDR correction.

^c^ Number of analyzed SNP within the gene or at +/- 50kb from the gene boundaries.

^d^ Per allele Odds Ratio (OR) for the top SNP at the gene locus.

**Supplementary Table S2.** Top ranked KEGG pathways (with *P*_EMP_<0.05).

| **KEGG Pathway** | **Number of genes in the pathway** | **Number of genes tagged by OncoArray SNPs** | ***P*_EMP_^a^** | ***P*_FDR_^b^** |
| --- | --- | --- | --- | --- |
| Cholesterol metabolism | 49 | 43 | 1.5 x 10^-3^ | 0.86 |
| Amino sugar and nucleotide sugar metabolism (*NANS*)* | 47 | 45 | 1.30 x 10^-2^ | 0.86 |
| Steroid biosynthesis | 17 | 17 | 1.30 x 10^-2^ | 0.86 |
| Cellular senescence | 154 | 135 | 1.40 x 10^-2^ | 0.86 |
| Taurine and hypotaurine metabolism | 11 | 10 | 1.70 x 10^-2^ | 0.86 |
| Thermogenesis | 193 | 164 | 1.90 x 10^-2^ | 0.86 |
| Cell cycle | 121 | 115 | 2.40 x 10^-2^ | 0.86 |
| Glyoxylate and dicarboxylate metabolism | 30 | 30 | 2.50 x 10^-2^ | 0.86 |
| Messenger RNA biogenesis (*ANP32B*) | 408 | 303 | 2.70 x 10^-2^ | 0.86 |
| MAPK signaling pathway | 284 | 222 | 3.10 x 10^-2^ | 0.86 |
| Staphylococcus aureus infection | 52 | 29 | 3.10 x 10^-2^ | 0.86 |
| EGFR tyrosine kinase inhibitor resistance (*NRG1*)* | 78 | 77 | 3.70 x 10^-2^ | 0.86 |
| Human T-cell leukemia virus 1 infection | 247 | 195 | 3.90 x 10^-2^ | 0.86 |
| Insulin resistance | 102 | 94 | 3.90 x 10^-2^ | 0.86 |
| One carbon pool by folate | 19 | 18 | 3.90 x 10^-2^ | 0.86 |
| Glycosaminoglycan degradation | 18 | 15 | 4.30 x 10^-2^ | 0.86 |
| Adipocytokine signaling pathway | 66 | 64 | 4.60 x 10^-2^ | 0.86 |
| Carbohydrate digestion and absorption | 41 | 35 | 4.70 x 10^-2^ | 0.86 |
| Fat digestion and absorption | 40 | 31 | 4.80 x 10^-2^ | 0.86 |
| Glycine, serine and threonine metabolism | 36 | 31 | 4.80 x 10^-2^ | 0.86 |
| Transfer RNA biogenesis (*TRMO*)* | 153 | 138 | 4.99 x 10^-2^ | 0.86 |

^a^ Empirical p-value of the association test at the gene level.

^b^ p-value of the association test with DTC risk at the gene level, after FDR correction.

* Pathway no longer associated with DTC after excluding significantly associated genes identified in the gene-level analysis.

When present in the pathway, significant genes from the gene-level analysis are indicated in brackets.

**Supplementary Table S3.** Top ranked Reactome pathways (with *P*_EMP_<0.05).

| **Reactome pathway** | **Number of genes in the pathway** | **Number of genes tagged by OncoArray SNPs** | ***P*_EMP_^a^** | ***P*_FDR_^b^** |
| --- | --- | --- | --- | --- |
| NCAM1 interactions | 36 | 31 | 7.20 x 10^-04^ | 0.93 |
| Downregulation of ERBB2:ERBB3 signaling (*NRG1*)* | 13 | 12 | 1.50 x 10^-03^ | 0.93 |
| Downregulation of ERBB2 signaling (*NRG1*)* | 29 | 26 | 2.30 x 10^-03^ | 0.93 |
| GRB7 events in ERBB2 signaling (*NRG1*)* | 5 | 4 | 2.50 x 10^-03^ | 0.93 |
| Import of palmitoyl CoA into the mitochondrial matrix | 11 | 11 | 2.80 x 10^-03^ | 0.93 |
| VEGFR2 mediated vascular permeability | 26 | 25 | 3.80 x 10^-03^ | 0.93 |
| Regulation of Insulin like Growth Factor (IGF) transport and uptake by Insulin like Growth Factor Binding Proteins (IGFBPs) | 119 | 100 | 4.40 x 10^-03^ | 0.93 |
| PI3K events in ERBB4 signaling (*NRG1*)* | 10 | 8 | 4.90 x 10^-03^ | 0.93 |
| TFAP2 (AP2) family regulates transcription of cell cycle factors | 5 | 5 | 6.70 x 10^-03^ | 0.93 |
| ERBB2 Activates PTK6 Signaling (*NRG1*)* | 13 | 11 | 7.60 x 10^-03^ | 0.93 |
| Signaling by ERBB2 (*NRG1*)* | 50 | 46 | 7.80 x 10^-03^ | 0.93 |
| STING mediated induction of host immune responses | 14 | 14 | 9.00 x 10^-03^ | 0.93 |
| Posttranslational protein phosphorylation | 102 | 88 | 9.60 x 10^-03^ | 0.93 |
| ERBB2 Regulates Cell Motility (*NRG1*)* | 15 | 13 | 1.10 x 10^-02^ | 0.93 |
| Activation of PPARGC1A (PGC1alpha) by phosphorylation | 10 | 9 | 1.20 x 10^-02^ | 0.93 |
| NCAM signaling for neurite outgrowth | 57 | 52 | 1.20 x 10^-02^ | 0.93 |
| Energy dependent regulation of mTOR by LKB1AMPK | 28 | 26 | 1.30 x 10^-02^ | 0.93 |
| IRF3mediated induction of type I IFN | 12 | 12 | 1.40 x 10^-02^ | 0.93 |
| Digestion of dietary lipid | 6 | 4 | 1.60 x 10^-02^ | 0.93 |
| Interaction between L1 and Ankyrins | 28 | 21 | 1.60 x 10^-02^ | 0.93 |
| Nuclear signaling by ERBB4 (*NRG1*)* | 27 | 25 | 1.60 x 10^-02^ | 0.93 |
| Signaling by VEGF | 99 | 96 | 1.60 x 10^-02^ | 0.93 |
| Resolution of D loop Structures through Holliday Junction Intermediates | 31 | 30 | 1.80 x 10^-02^ | 0.93 |
| PI3K events in ERBB2 signaling (*NRG1*)* | 16 | 14 | 1.90 x 10^-02^ | 0.93 |
| SLBP independent Processing of Histone Prem RNAs | 10 | 10 | 1.90 x 10^-02^ | 0.93 |
| Insulin like Growth Factor2 mRNA Binding Proteins (IGF2BPs/IMPs/VICKZs) bind RNA | 7 | 7 | 2.00 x 10^-02^ | 0.93 |
| PTEN Regulation | 137 | 120 | 2.00 x 10^-02^ | 0.93 |
| SLBP Dependent Processing of Replication Dependent Histone Prem RNAs | 11 | 11 | 2.10 x 10^-02^ | 0.93 |
| mTOR signaling | 39 | 36 | 2.20 x 10^-02^ | 0.93 |
| NrCAM interactions | 6 | 6 | 2.27 x 10^-02^ | 0.93 |
| Cyclin E associated events during G1/S transition | 82 | 78 | 2.30 x 10^-02^ | 0.93 |
| Multifunctional anion exchangers | 9 | 8 | 2.30 x 10^-02^ | 0.93 |
| Intracellular signaling by second messengers (*NRG1*)* | 284 | 236 | 2.60 x 10^-02^ | 0.93 |
| MAPK6/MAPK4 signaling | 90 | 80 | 2.60 x 10^-02^ | 0.93 |
| Cyclin A:Cdk2associated events at S phase entry | 84 | 80 | 2.80 x 10^-02^ | 0.93 |
| SHC1 events in ERBB4 signaling (*NRG1*)* | 14 | 12 | 2.80 x 10^-02^ | 0.93 |
| tRNA modification in the mitochondrion | 8 | 8 | 2.90 x 10^-02^ | 0.93 |
| Cholesterol biosynthesis | 23 | 21 | 3.00 x 10^-02^ | 0.93 |
| Resolution of D Loop Structures | 32 | 31 | 3.00 x 10^-02^ | 0.93 |
| AURKA Activation by TPX2 | 69 | 65 | 3.10 x 10^-02^ | 0.93 |
| FGFR1 mutant receptor activation | 31 | 26 | 3.10 x 10^-02^ | 0.93 |
| GRB2 events in ERBB2 signaling (*NRG1*)* | 16 | 14 | 3.10 x 10^-02^ | 0.93 |
| VEGF ligand receptor interactions | 7 | 7 | 3.20 x 10^-02^ | 0.93 |
| Chylomicron assembly | 10 | 7 | 3.30 x 10^-02^ | 0.93 |
| Synthesis of PG | 8 | 8 | 3.30 x 10^-02^ | 0.93 |
| L1CAM interactions | 92 | 79 | 3.40 x 10^-02^ | 0.93 |
| Regulation of PLK1 Activity at G2/M Transition | 84 | 79 | 3.50 x 10^-02^ | 0.93 |
| Signaling by Non Receptor Tyrosine Kinases (*NRG1*)* | 54 | 50 | 3.50 x 10^-02^ | 0.93 |
| Metabolism of proteins (*NANS*)* | 1902 | 832 | 3.60 x 10^-02^ | 0.93 |
| Uptake and function of anthrax toxins | 11 | 11 | 3.60 x 10^-02^ | 0.93 |
| Centrosome maturation | 78 | 72 | 3.70 x 10^-02^ | 0.93 |
| Noncanonical activation of NOTCH3 | 8 | 8 | 3.70 x 10^-02^ | 0.93 |
| Activation of NIMA Kinases NEK9, NEK6, NEK7 | 7 | 7 | 3.80 x 10^-02^ | 0.93 |
| Regulation of PTEN gene transcription | 59 | 53 | 3.80 x 10^-02^ | 0.93 |
| G2/M Transition | 178 | 158 | 4.20 x 10^-02^ | 0.93 |
| SHC1 events in ERBB2 signaling (*NRG1*)* | 22 | 20 | 4.20 x 10^-02^ | 0.93 |
| Disease (*NRG1*)* | 824 | 540 | 4.30 x 10^-02^ | 0.93 |
| Alpha defensins | 8 | 4 | 4.40 x 10^-02^ | 0.93 |
| Recruitment of NuMA to mitotic centrosomes | 77 | 72 | 4.40 x 10^-02^ | 0.93 |
| Sema4D mediated inhibition of cell attachment and migration | 8 | 8 | 4.40 x 10^-02^ | 0.93 |
| Betaoxidation of pristanoyl CoA | 9 | 9 | 4.50 x 10^-02^ | 0.93 |
| Loss of proteins required for interphase microtubule organization from the centrosome | 66 | 62 | 4.50 x 10^-02^ | 0.93 |
| Signaling by PTK6 (*NRG1*)* | 54 | 50 | 4.50 x 10^-02^ | 0.93 |
| Cell Cycle | 1052 | 412 | 4.60 x 10^-02^ | 0.93 |
| Vitamin C (ascorbate) metabolism | 8 | 7 | 4.70 x 10^-02^ | 0.93 |
| Amyloid fiber formation | 76 | 56 | 4.80 x 10^-02^ | 0.93 |
| Anchoring of the basal body to the plasma membrane | 93 | 87 | 4.80 x 10^-02^ | 0.93 |
| Neurofascin interactions | 6 | 6 | 4.80 x 10^-02^ | 0.93 |
| The role of GTSE1 in G2/M progression after G2 checkpoint | 59 | 55 | 4.80 x 10^-02^ | 0.93 |
| HDR through Homologous Recombination (HRR) | 65 | 63 | 4.90 x 10^-02^ | 0.93 |
| RUNX3 regulates BCL2L11 (BIM) transcription | 5 | 5 | 4.90 x 10^-02^ | 0.93 |
| Signaling by cytosolic FGFR1 fusion mutants | 18 | 16 | 4.90 x 10^-02^ | 0.93 |
| VEGF binds to VEGFR leading to receptor dimerization | 7 | 7 | 4.90 x 10^-02^ | 0.93 |
| Processing of Capped Intronless Prem RNA | 27 | 27 | 5.00 x 10^-02^ | 0.93 |
| SCF(Skp2)-mediated degradation of p27/p21 | 59 | 56 | 5.00 x 10^-02^ | 0.93 |

a Empirical p-value of the association test with DTC risk at the pathway level.

^b^ p-value of the association test with DTC risk at the pathway level, after FDR correction.

* Pathway no longer associated with DTC after excluding significantly associated genes identified in the gene-level analysis. When present in the pathways, significant genes from the gene-level analysis are indicated in brackets.

**Supplementary Table S4.** Top ranked GO terms (with *P*_EMP_<0.05).

| **GO category** | **GO Definition** | **Number of genes in the pathway** | **Number of genes tagged by OncoArray SNPs** | ***P*_EMP_^a^** | ***P*_FDR_^b^** |
| --- | --- | --- | --- | --- | --- |
| ***Biological processes*** | |  |  |  |  |
|  | Fatty acid transmembrane transport | 15 | 15 | 5.40 x 10^-04^ | 0.73 |
|  | Molting cycle process | 87 | 84 | 9.60 x 10^-04^ | 0.73 |
|  | Hair cycle process | 87 | 84 | 1.06 x 10^-03^ | 0.73 |
|  | Epidermis morphogenesis | 29 | 28 | 1.08 x 10^-03^ | 0.73 |
|  | GTP metabolic process | 31 | 29 | 1.10 x 10^-03^ | 0.73 |
|  | Carnitine shuttle | 10 | 10 | 1.10 x 10^-03^ | 0.73 |
|  | Secondary palate development | 10 | 10 | 1.18 x 10^-03^ | 0.73 |
|  | Skin epidermis development | 87 | 84 | 1.30 x 10^-03^ | 0.73 |
|  | Ventricular trabecula myocardium morphogenesis | 16 | 16 | 1.40 x 10^-03^ | 0.73 |
|  | Negative regulation of protein serine/threonine kinase activity | 127 | 119 | 1.50 x 10^-03^ | 0.73 |
|  | Anatomical structure maturation | 145 | 130 | 1.60 x 10^-03^ | 0.73 |
|  | Regulation of cardiocyte differentiation | 40 | 40 | 1.70 x 10^-03^ | 0.73 |
|  | Regulation of cardiac muscle cell differentiation | 28 | 28 | 1.90 x 10^-03^ | 0.73 |
|  | Regulation of cell cycle G2/M phase transition | 196 | 173 | 2.10 x 10^-03^ | 0.73 |
|  | Neuron maturation | 39 | 37 | 2.30 x 10^-03^ | 0.73 |
|  | Endocardium development | 11 | 11 | 2.70 x 10^-03^ | 0.73 |
|  | Regulation of fatty acid oxidation | 28 | 28 | 2.70 x 10^-03^ | 0.73 |
|  | Peripheral nervous system development | 76 | 71 | 2.80 x 10^-03^ | 0.73 |
|  | Longchain fatty acid transport | 60 | 55 | 2.80 x 10^-03^ | 0.73 |
|  | Regulation of protein heterodimerization activity | 10 | 10 | 2.80 x 10^-03^ | 0.73 |
|  | Cardioblast differentiation | 17 | 17 | 3.10 x 10^-03^ | 0.73 |
|  | Negative regulation of neuron migration | 9 | 9 | 3.20 x 10^-03^ | 0.73 |
|  | Cardiac conduction system development | 13 | 12 | 3.60 x 10^-03^ | 0.73 |
|  | Positive regulation of intracellular protein transport | 180 | 164 | 3.70 x 10^-03^ | 0.73 |
|  | Positive regulation of myelination | 12 | 12 | 3.90 x 10^-03^ | 0.73 |
|  | Calcineurinmediated signaling | 30 | 30 | 3.90 x 10^-03^ | 0.73 |
|  | Positive regulation of cardiocyte differentiation | 25 | 25 | 4.00 x 10^-03^ | 0.73 |
|  | Regulation of intracellular protein transport | 244 | 214 | 4.20 x 10^-03^ | 0.73 |
|  | Guanosinecontaining compound metabolic process | 48 | 45 | 4.20 x 10^-03^ | 0.73 |
|  | Mitochondrial transport | 284 | 240 | 4.50 x 10^-03^ | 0.73 |
|  | Molting cycle | 108 | 95 | 4.50 x 10^-03^ | 0.73 |
|  | Protein localization to mitochondrion | 181 | 165 | 4.50 x 10^-03^ | 0.73 |
|  | Purine nucleoside metabolic process | 71 | 67 | 5.00 x 10^-03^ | 0.75 |
|  | Cardiac muscle cell myoblast differentiation | 10 | 10 | 5.00 x 10^-03^ | 0.75 |
|  | Thyroid hormone generation | 16 | 13 | 5.20 x 10^-03^ | 0.75 |
|  | Regulation of MAP kinase activity | 323 | 273 | 5.20 x 10^-03^ | 0.75 |
|  | Thyroid hormone metabolic process | 20 | 17 | 5.40 x 10^-03^ | 0.76 |
|  | Glial cell fate commitment | 13 | 13 | 5.80 x 10^-03^ | 0.78 |
|  | Positive regulation of cardiac muscle cell differentiation | 17 | 17 | 6.00 x 10^-03^ | 0.78 |
|  | Tetrahydrofolate interconversion | 9 | 9 | 6.30 x 10^-03^ | 0.78 |
|  | Hair cycle | 108 | 95 | 6.30 x 10^-03^ | 0.78 |
|  | Positive regulation of nucleocytoplasmic transport | 55 | 53 | 6.30 x 10^-03^ | 0.78 |
|  | Activation of transmembrane receptor protein tyrosine kinase activity | 13 | 13 | 6.50 x 10^-03^ | 0.79 |
|  | Positive regulation of protein localization to cell surface | 13 | 13 | 7.00 x 10^-03^ | 0.83 |
|  | Regulation of RNA binding | 9 | 9 | 7.20 x 10^-03^ | 0.83 |
|  | Positive regulation of neurological system process | 23 | 23 | 7.30 x 10^-03^ | 0.83 |
|  | Regulation of nucleocytoplasmic transport | 99 | 94 | 8.00 x 10^-03^ | 0.88 |
|  | Activation of protein kinase B activity | 32 | 31 | 9.60 x 10^-03^ | 0.88 |
|  | Cell proliferation in hindbrain | 11 | 11 | 1.00 x 10^-02^ | 0.88 |
|  | Locomotory behavior | 184 | 172 | 1.04 x 10^-02^ | 0.88 |
|  | Inositol phosphatemediated signaling | 39 | 39 | 1.04 x 10^-02^ | 0.88 |
|  | RNA modification | 133 | 121 | 1.09 x 10^-02^ | 0.88 |
|  | Activation of protein kinase activity | 312 | 269 | 1.10 x 10^-02^ | 0.88 |
|  | Intracellular lipid transport | 31 | 30 | 1.10 x 10^-02^ | 0.88 |
|  | Trabecula morphogenesis | 49 | 48 | 1.10 x 10^-02^ | 0.88 |
|  | Ensheathment of neurons | 112 | 104 | 1.11 x 10^-02^ | 0.88 |
|  | Ribonucleoside metabolic process | 95 | 86 | 1.20 x 10^-02^ | 0.88 |
|  | Regulation of protein homodimerization activity | 19 | 19 | 1.20 x 10^-02^ | 0.88 |
|  | Cell cycle G2/M phase transition | 246 | 216 | 1.20 x 10^-02^ | 0.88 |
|  | Regulation of sphingolipid biosynthetic process | 14 | 14 | 1.20 x 10^-02^ | 0.88 |
|  | Trna methylation | 30 | 30 | 1.20 x 10^-02^ | 0.88 |
|  | Neuron fate commitment | 67 | 63 | 1.24 x 10^-02^ | 0.88 |
|  | Axon ensheathment | 112 | 104 | 1.30 x 10^-02^ | 0.88 |
|  | Sterol biosynthetic process | 69 | 63 | 1.30 x 10^-02^ | 0.88 |
|  | Thyroid gland development | 25 | 23 | 1.30 x 10^-02^ | 0.88 |
|  | Positive regulation of chromatin binding | 13 | 12 | 1.30 x 10^-02^ | 0.88 |
|  | Hair follicle maturation | 16 | 16 | 1.30 x 10^-02^ | 0.88 |
|  | Meiotic cell cycle | 226 | 196 | 1.30 x 10^-02^ | 0.88 |
|  | Positive regulation of dendrite development | 62 | 62 | 1.30 x 10^-02^ | 0.88 |
|  | Regulation of ceramide biosynthetic process | 14 | 14 | 1.30 x 10^-02^ | 0.88 |
|  | Regulation of fatty acid betaoxidation | 16 | 16 | 1.32 x 10^-02^ | 0.88 |
|  | Ventricular cardiac muscle tissue development | 54 | 51 | 1.40 x 10^-02^ | 0.88 |
|  | Schwann cell differentiation | 36 | 33 | 1.40 x 10^-02^ | 0.88 |
|  | TORC1 signaling | 32 | 31 | 1.40 x 10^-02^ | 0.88 |
|  | Actin filament reorganization | 10 | 10 | 1.40 x 10^-02^ | 0.88 |
|  | Cellular response to environmental stimulus | 293 | 249 | 1.40 x 10^-02^ | 0.88 |
|  | Fatty acid betaoxidation | 71 | 68 | 1.50 x 10^-02^ | 0.88 |
|  | Fatty acid transport | 84 | 78 | 1.50 x 10^-02^ | 0.88 |
|  | Developmental maturation | 246 | 208 | 1.50 x 10^-02^ | 0.88 |
|  | Forebrain cell migration | 54 | 53 | 1.50 x 10^-02^ | 0.88 |
|  | Central nervous system neuron axonogenesis | 29 | 29 | 1.50 x 10^-02^ | 0.88 |
|  | Regulation of myelination | 34 | 33 | 1.50 x 10^-02^ | 0.88 |
|  | Positive regulation of extrinsic apoptotic signaling pathway via death domain receptors | 17 | 17 | 1.50 x 10^-02^ | 0.88 |
|  | Startle response | 24 | 24 | 1.60 x 10^-02^ | 0.88 |
|  | Plasma membrane organization | 75 | 66 | 1.60 x 10^-02^ | 0.88 |
|  | Sterol metabolic process | 147 | 130 | 1.60 x 10^-02^ | 0.88 |
|  | Intras DNA damage checkpoint | 14 | 14 | 1.60 x 10^-02^ | 0.88 |
|  | Cardiac muscle tissue morphogenesis | 67 | 63 | 1.60 x 10^-02^ | 0.88 |
|  | Cellular response to abiotic stimulus | 293 | 249 | 1.60 x 10^-02^ | 0.88 |
|  | Regulation of membrane lipid metabolic process | 14 | 14 | 1.60 x 10^-02^ | 0.88 |
|  | Postembryonic development | 87 | 84 | 1.60 x 10^-02^ | 0.88 |
|  | Positive regulation of intracellular transport | 225 | 203 | 1.61 x 10^-02^ | 0.88 |
|  | Regulation of transcription by RNA polymerase III | 25 | 25 | 1.70 x 10^-02^ | 0.88 |
|  | Protein targeting to mitochondrion | 139 | 130 | 1.70 x 10^-02^ | 0.88 |
|  | Regulation of ARF protein signal transduction | 42 | 41 | 1.70 x 10^-02^ | 0.88 |
|  | Heart trabecula morphogenesis | 34 | 34 | 1.70 x 10^-02^ | 0.88 |
|  | Mitochondrial RNA processing | 15 | 15 | 1.80 x 10^-02^ | 0.88 |
|  | Phosphatidylcholine biosynthetic process | 33 | 32 | 1.80 x 10^-02^ | 0.88 |
|  | Adult locomotory behavior | 77 | 73 | 1.80 x 10^-02^ | 0.88 |
|  | Regulation of fatty acid metabolic process | 77 | 73 | 1.80 x 10^-02^ | 0.88 |
|  | Dopamine biosynthetic process | 11 | 11 | 1.80 x 10^-02^ | 0.88 |
|  | Lipoprotein transport | 14 | 14 | 1.80 x 10^-02^ | 0.88 |
|  | Positive regulation of transcription by RNA polymerase III | 10 | 10 | 1.80 x 10^-02^ | 0.88 |
|  | Muscle organ morphogenesis | 84 | 79 | 1.80 x 10^-02^ | 0.88 |
|  | Oligodendrocyte differentiation | 85 | 81 | 1.80 x 10^-02^ | 0.88 |
|  | Quaternary ammonium group transport | 13 | 10 | 1.90 x 10^-02^ | 0.88 |
|  | Telencephalon regionalization | 13 | 13 | 1.90 x 10^-02^ | 0.88 |
|  | Sodium ion export across plasma membrane | 14 | 12 | 1.90 x 10^-02^ | 0.88 |
|  | Positive regulation of cell cycle G2/M phase transition | 25 | 25 | 1.90 x 10^-02^ | 0.88 |
|  | Regulation of protein localization to cell surface | 33 | 33 | 1.90 x 10^-02^ | 0.88 |
|  | Schwann cell development | 28 | 25 | 2.00 x 10^-02^ | 0.88 |
|  | Positive regulation of telomerase activity | 35 | 34 | 2.00 x 10^-02^ | 0.88 |
|  | Negative regulation of lipase activity | 15 | 14 | 2.00 x 10^-02^ | 0.88 |
|  | Positive regulation of cellular protein localization | 326 | 274 | 2.00 x 10^-02^ | 0.88 |
|  | Glycine metabolic process | 12 | 12 | 2.10 x 10^-02^ | 0.88 |
|  | Cell cycle arrest | 241 | 207 | 2.10 x 10^-02^ | 0.88 |
|  | Protein localization to cell surface | 51 | 51 | 2.10 x 10^-02^ | 0.88 |
|  | Lipid oxidation | 100 | 93 | 2.10 x 10^-02^ | 0.88 |
|  | Thymus development | 45 | 44 | 2.10 x 10^-02^ | 0.88 |
|  | Regulation of striated muscle cell differentiation | 90 | 86 | 2.10 x 10^-02^ | 0.88 |
|  | Cellular response to light stimulus | 109 | 96 | 2.10 x 10^-02^ | 0.88 |
|  | Female meiotic nuclear division | 27 | 27 | 2.20 x 10^-02^ | 0.88 |
|  | Potassium ion import | 40 | 33 | 2.20 x 10^-02^ | 0.88 |
|  | Lipoprotein localization | 14 | 14 | 2.20 x 10^-02^ | 0.88 |
|  | Tetrahydrofolate metabolic process | 18 | 18 | 2.20 x 10^-02^ | 0.88 |
|  | Negative regulation of small gtpase mediated signal transduction | 52 | 51 | 2.20 x 10^-02^ | 0.88 |
|  | Regulation of cardiac muscle tissue development | 68 | 68 | 2.20 x 10^-02^ | 0.88 |
|  | Carboxylic acid transmembrane transport | 111 | 98 | 2.20 x 10^-02^ | 0.88 |
|  | Mitochondrial transmembrane transport | 69 | 66 | 2.20 x 10^-02^ | 0.88 |
|  | Cellular response to leukemia inhibitory factor | 92 | 91 | 2.20 x 10^-02^ | 0.88 |
|  | Histone phosphorylation | 34 | 33 | 2.30 x 10^-02^ | 0.89 |
|  | Negative regulation of tyrosine phosphorylation of STAT protein | 11 | 11 | 2.30 x 10^-02^ | 0.89 |
|  | Pyrimidine ribonucleoside metabolic process | 30 | 25 | 2.30 x 10^-02^ | 0.89 |
|  | Organic hydroxy compound biosynthetic process | 231 | 208 | 2.30 x 10^-02^ | 0.89 |
|  | Trna modification | 73 | 71 | 2.40 x 10^-02^ | 0.92 |
|  | Glyceraldehyde3phosphate metabolic process | 22 | 22 | 2.40 x 10^-02^ | 0.92 |
|  | Histone mrna metabolic process | 22 | 22 | 2.50 x 10^-02^ | 0.92 |
|  | ARF protein signal transduction | 42 | 41 | 2.50 x 10^-02^ | 0.92 |
|  | Antigen processing and presentation of exogenous peptide antigen via MHC class I | 72 | 61 | 2.50 x 10^-02^ | 0.92 |
|  | Regulation of calcineurinnfat signaling cascade | 17 | 17 | 2.50 x 10^-02^ | 0.92 |
|  | Response to angiotensin | 22 | 22 | 2.50 x 10^-02^ | 0.92 |
|  | Activation of MAPK activity | 139 | 129 | 2.60 x 10^-02^ | 0.92 |
|  | Regulation of phospholipase A2 activity | 10 | 10 | 2.60 x 10^-02^ | 0.92 |
|  | Interstrand crosslink repair | 46 | 45 | 2.60 x 10^-02^ | 0.92 |
|  | Response to mercury ion | 10 | 10 | 2.60 x 10^-02^ | 0.92 |
|  | Positive regulation of lipid catabolic process | 22 | 20 | 2.60 x 10^-02^ | 0.92 |
|  | Regulation of cyclindependent protein kinase activity | 86 | 79 | 2.60 x 10^-02^ | 0.92 |
|  | Meiotic spindle organization | 12 | 11 | 2.70 x 10^-02^ | 0.94 |
|  | Nucleoside metabolic process | 113 | 102 | 2.70 x 10^-02^ | 0.94 |
|  | Positive regulation of G2/M transition of mitotic cell cycle | 21 | 21 | 2.70 x 10^-02^ | 0.94 |
|  | Pulmonary valve morphogenesis | 12 | 12 | 2.80 x 10^-02^ | 0.94 |
|  | Phenolcontaining compound metabolic process | 85 | 79 | 2.80 x 10^-02^ | 0.94 |
|  | Locomotor rhythm | 12 | 12 | 2.80 x 10^-02^ | 0.94 |
|  | Negative regulation of cell cycle G2/M phase transition | 94 | 87 | 2.80 x 10^-02^ | 0.94 |
|  | Organic acid transmembrane transport | 111 | 98 | 2.80 x 10^-02^ | 0.94 |
|  | Pulmonary valve development | 12 | 12 | 2.90 x 10^-02^ | 0.95 |
|  | Negative chemotaxis | 35 | 32 | 2.90 x 10^-02^ | 0.95 |
|  | Regulation of calcineurinmediated signaling | 17 | 17 | 2.90 x 10^-02^ | 0.95 |
|  | Regulation of TORC1 signaling | 25 | 25 | 2.90 x 10^-02^ | 0.95 |
|  | Lipid digestion | 15 | 12 | 3.00 x 10^-02^ | 0.97 |
|  | Positive regulation of striated muscle cell differentiation | 54 | 53 | 3.00 x 10^-02^ | 0.97 |
|  | Pyrimidine ribonucleotide metabolic process | 21 | 20 | 3.10 x 10^-02^ | 0.97 |
|  | Sterol transport | 84 | 77 | 3.10 x 10^-02^ | 0.97 |
|  | UTP metabolic process | 10 | 9 | 3.10 x 10^-02^ | 0.97 |
|  | Cell adhesion molecule production | 7 | 7 | 3.10 x 10^-02^ | 0.97 |
|  | Mitochondrial trna processing | 11 | 11 | 3.10 x 10^-02^ | 0.97 |
|  | Steroid biosynthetic process | 178 | 155 | 3.20 x 10^-02^ | 0.98 |
|  | Response to leukemia inhibitory factor | 92 | 91 | 3.20 x 10^-02^ | 0.98 |
|  | Negative regulation of campdependent protein kinase activity | 8 | 8 | 3.20 x 10^-02^ | 0.98 |
|  | SMAD protein complex assembly | 14 | 13 | 3.30 x 10^-02^ | 0.98 |
|  | Nucleotidesugar biosynthetic process | 22 | 22 | 3.30 x 10^-02^ | 0.98 |
|  | Cellular response to UV | 72 | 64 | 3.30 x 10^-02^ | 0.98 |
|  | Positive regulation of vascular permeability | 10 | 10 | 3.30 x 10^-02^ | 0.98 |
|  | Fibroblast apoptotic process | 18 | 18 | 3.30 x 10^-02^ | 0.98 |
|  | Regulation of neutrophil chemotaxis | 32 | 23 | 3.30 x 10^-02^ | 0.98 |
|  | Negative regulation of cytokine production involved in immune response | 23 | 21 | 3.40 x 10^-02^ | 0.99 |
|  | Fatty acid catabolic process | 108 | 102 | 3.40 x 10^-02^ | 0.99 |
|  | Positive regulation of cell adhesion mediated by integrin | 17 | 17 | 3.40 x 10^-02^ | 0.99 |
|  | DNA doublestrand break processing | 23 | 23 | 3.50 x 10^-02^ | 0.99 |
|  | Pentosephosphate shunt | 17 | 17 | 3.50 x 10^-02^ | 0.99 |
|  | Pyrimidine nucleotide metabolic process | 42 | 40 | 3.50 x 10^-02^ | 0.99 |
|  | Coenzyme metabolic process | 360 | 287 | 3.50 x 10^-02^ | 0.99 |
|  | Regulation of telomere maintenance | 70 | 65 | 3.50 x 10^-02^ | 0.99 |
|  | Regulation of protein import | 53 | 50 | 3.50 x 10^-02^ | 0.99 |
|  | Negative regulation of MAP kinase activity | 71 | 69 | 3.60 x 10^-02^ | 1.00 |
|  | Negative regulation of cell division | 16 | 16 | 3.60 x 10^-02^ | 1.00 |
|  | Trna threonylcarbamoyladenosine metabolic process | 12 | 12 | 3.60 x 10^-02^ | 1.00 |
|  | Negative regulation of insulinlike growth factor receptor signaling pathway | 7 | 7 | 3.70 x 10^-02^ | 1.00 |
|  | Organelle inheritance | 15 | 15 | 3.70 x 10^-02^ | 1.00 |
|  | Regulation of phosphorylation of RNA polymerase II Cterminal domain | 9 | 9 | 3.70 x 10^-02^ | 1.00 |
|  | RNA methylation | 57 | 56 | 3.80 x 10^-02^ | 1.00 |
|  | Doublestrand break repair | 202 | 172 | 3.80 x 10^-02^ | 1.00 |
|  | Protein autophosphorylation | 223 | 196 | 3.80 x 10^-02^ | 1.00 |
|  | Peptidyltyrosine modification | 365 | 280 | 3.90 x 10^-02^ | 1.00 |
|  | Fatty acid betaoxidation using acylcoa oxidase | 14 | 14 | 3.90 x 10^-02^ | 1.00 |
|  | Gliogenesis | 253 | 225 | 3.90 x 10^-02^ | 1.00 |
|  | Positive regulation of neutrophil migration | 32 | 23 | 3.90 x 10^-02^ | 1.00 |
|  | Inactivation of MAPK activity | 24 | 24 | 4.00 x 10^-02^ | 1.00 |
|  | Establishment or maintenance of transmembrane electrochemical gradient | 13 | 12 | 4.00 x 10^-02^ | 1.00 |
|  | Animal organ regeneration | 73 | 70 | 4.00 x 10^-02^ | 1.00 |
|  | Cellular response to radiation | 167 | 147 | 4.00 x 10^-02^ | 1.00 |
|  | Response to peptidoglycan | 10 | 10 | 4.10 x 10^-02^ | 1.00 |
|  | Mitochondrial protein processing | 12 | 12 | 4.10 x 10^-02^ | 1.00 |
|  | Mrna transcription by RNA polymerase II | 18 | 18 | 4.10 x 10^-02^ | 1.00 |
|  | Cell maturation | 157 | 143 | 4.10 x 10^-02^ | 1.00 |
|  | Regulation of protein targeting | 117 | 109 | 4.10 x 10^-02^ | 1.00 |
|  | Regulation of stem cell differentiation | 139 | 110 | 4.10 x 10^-02^ | 1.00 |
|  | Negative regulation of production of molecular mediator of immune response | 31 | 28 | 4.20 x 10^-02^ | 1.00 |
|  | Membrane disruption in other organism | 10 | 5 | 4.20 x 10^-02^ | 1.00 |
|  | Intestinal lipid absorption | 15 | 12 | 4.20 x 10^-02^ | 1.00 |
|  | Trna processing | 116 | 110 | 4.30 x 10^-02^ | 1.00 |
|  | Positive regulation of phosphatidylinositol 3kinase signaling | 65 | 58 | 4.30 x 10^-02^ | 1.00 |
|  | Regulation of intracellular transport | 371 | 314 | 4.30 x 10^-02^ | 1.00 |
|  | Lserine metabolic process | 12 | 10 | 4.40 x 10^-02^ | 1.00 |
|  | Pyrimidine nucleoside monophosphate metabolic process | 15 | 15 | 4.40 x 10^-02^ | 1.00 |
|  | Myotube cell development | 31 | 30 | 4.40 x 10^-02^ | 1.00 |
|  | Muscle cell development | 153 | 141 | 4.40 x 10^-02^ | 1.00 |
|  | Meiotic cell cycle process | 176 | 154 | 4.40 x 10^-02^ | 1.00 |
|  | Reciprocal meiotic recombination | 48 | 44 | 4.50 x 10^-02^ | 1.00 |
|  | Muscle fiber development | 52 | 50 | 4.50 x 10^-02^ | 1.00 |
|  | Positive regulation of type I interferonmediated signaling pathway | 11 | 11 | 4.50 x 10^-02^ | 1.00 |
|  | Regulation of phospholipase activity | 58 | 54 | 4.60 x 10^-02^ | 1.00 |
|  | Base conversion or substitution editing | 17 | 11 | 4.60 x 10^-02^ | 1.00 |
|  | Negative regulation of Wnt signaling pathway | 195 | 173 | 4.60 x 10^-02^ | 1.00 |
|  | Basement membrane organization | 20 | 20 | 4.60 x 10^-02^ | 1.00 |
|  | Ciliary basal bodyplasma membrane docking | 92 | 86 | 4.60 x 10^-02^ | 1.00 |
|  | Export across plasma membrane | 22 | 20 | 4.60 x 10^-02^ | 1.00 |
|  | Positive regulation of protein import | 34 | 33 | 4.60 x 10^-02^ | 1.00 |
|  | Regulation of microtubule motor activity | 11 | 11 | 4.60 x 10^-02^ | 1.00 |
|  | Regulation of neuron migration | 32 | 32 | 4.60 x 10^-02^ | 1.00 |
|  | Cardiac ventricle development | 117 | 113 | 4.70 x 10^-02^ | 1.00 |
|  | Carnitine metabolic process | 12 | 11 | 4.70 x 10^-02^ | 1.00 |
|  | Neuronal action potential | 33 | 26 | 4.70 x 10^-02^ | 1.00 |
|  | Lamellipodium assembly | 53 | 51 | 4.70 x 10^-02^ | 1.00 |
|  | Myelin maintenance | 14 | 12 | 4.70 x 10^-02^ | 1.00 |
|  | Regulation of meiotic cell cycle | 43 | 42 | 4.70 x 10^-02^ | 1.00 |
|  | Resolution of meiotic recombination intermediates | 13 | 11 | 4.80 x 10^-02^ | 1.00 |
|  | Cardiac ventricle morphogenesis | 72 | 69 | 4.80 x 10^-02^ | 1.00 |
|  | Termination of RNA polymerase II transcription | 66 | 65 | 4.80 x 10^-02^ | 1.00 |
|  | Muscle organ development | 352 | 304 | 4.80 x 10^-02^ | 1.00 |
|  | Positive regulation of transcription by RNA polymerase I | 19 | 19 | 4.80 x 10^-02^ | 1.00 |
|  | Positive regulation of cell junction assembly | 30 | 30 | 4.80 x 10^-02^ | 1.00 |
|  | Regulation of cellular ketone metabolic process | 156 | 139 | 4.90 x 10^-02^ | 1.00 |
|  | Ventricular system development | 23 | 23 | 4.90 x 10^-02^ | 1.00 |
|  | Regulation of lipid catabolic process | 50 | 46 | 4.90 x 10^-02^ | 1.00 |
|  | Regulation of cell maturation | 21 | 21 | 4.90 x 10^-02^ | 1.00 |
|  | Folic acidcontaining compound metabolic process | 28 | 25 | 5.00 x 10^-02^ | 1.00 |
|  | Positive regulation of telomere maintenance | 47 | 46 | 5.00 x 10^-02^ | 1.00 |
|  | Inositol phosphate biosynthetic process | 27 | 27 | 5.00 x 10^-02^ | 1.00 |
|  | Microtubule organizing center localization | 25 | 24 | 5.00 x 10^-02^ | 1.00 |
|  | Cellular response to osmotic stress | 31 | 31 | 5.00 x 10^-02^ | 1.00 |
|  | Activation of cysteinetype endopeptidase activity | 13 | 13 | 5.00 x 10^-02^ | 1.00 |
|  | Protein localization to microtubule organizing center | 27 | 27 | 5.00 x 10^-02^ | 1.00 |
|  |  |  |  |  |  |
| ***Molecular functions*** | |  |  |  |  |
|  | Nucleotideactivated protein kinase complex | 10 | 10 | 2.10 x 10^-03^ | 0.65 |
|  | Ada2/Gcn5/Ada3 transcription activator complex | 12 | 12 | 3.60 x 10^-03^ | 0.65 |
|  | Acrosomal vesicle | 107 | 94 | 3.90 x 10^-03^ | 0.65 |
|  | VCPNPL4UFD1 AAA atpase complex | 9 | 9 | 4.10 x 10^-03^ | 0.65 |
|  | Main axon | 63 | 59 | 6.10 x 10^-03^ | 0.65 |
|  | Sperm part | 177 | 150 | 7.20 x 10^-03^ | 0.65 |
|  | Protein kinase complex | 94 | 86 | 1.03 x 10^-02^ | 0.65 |
|  | Mediator complex | 34 | 33 | 1.10 x 10^-02^ | 0.65 |
|  | Axon initial segment | 12 | 12 | 1.10 x 10^-02^ | 0.65 |
|  | Nucleoid | 40 | 39 | 1.14 x 10^-02^ | 0.65 |
|  | Mitochondrial nucleoid | 40 | 39 | 1.24 x 10^-02^ | 0.65 |
|  | Sodium potassiumexchanging atpase complex | 10 | 9 | 1.30 x 10^-02^ | 0.65 |
|  | Node of Ranvier | 15 | 13 | 1.40 x 10^-02^ | 0.65 |
|  | Cationtransporting atpase complex | 14 | 13 | 1.50 x 10^-02^ | 0.65 |
|  | Nucleotideexcision repair complex | 9 | 9 | 1.60 x 10^-02^ | 0.65 |
|  | Proteasome accessory complex | 23 | 22 | 1.60 x 10^-02^ | 0.65 |
|  | Vesicle lumen | 322 | 251 | 1.80 x 10^-02^ | 0.69 |
|  | MKS complex | 13 | 13 | 2.10 x 10^-02^ | 0.72 |
|  | Atpase complex | 93 | 84 | 2.10 x 10^-02^ | 0.72 |
|  | Proteinlipid complex | 39 | 31 | 2.20 x 10^-02^ | 0.72 |
|  | Axolemma | 16 | 15 | 2.50 x 10^-02^ | 0.74 |
|  | Palmitoyltransferase complex | 10 | 10 | 2.60 x 10^-02^ | 0.74 |
|  | Atpase dependent transmembrane transport complex | 22 | 19 | 2.60 x 10^-02^ | 0.74 |
|  | ESC/E(Z) complex | 17 | 17 | 3.00 x 10^-02^ | 0.81 |
|  | H4 histone acetyltransferase complex | 36 | 36 | 3.30 x 10^-02^ | 0.82 |
|  | Clathrincoated pit | 65 | 62 | 3.40 x 10^-02^ | 0.82 |
|  | Iswitype complex | 10 | 10 | 3.60 x 10^-02^ | 0.82 |
|  | Triglyceriderich plasma lipoprotein particle | 18 | 12 | 3.70 x 10^-02^ | 0.82 |
|  | Platelet dense granule | 19 | 18 | 3.70 x 10^-02^ | 0.82 |
|  | Ttubule | 45 | 42 | 3.80 x 10^-02^ | 0.82 |
|  | DNA repair complex | 34 | 33 | 3.90 x 10^-02^ | 0.82 |
|  | Intrinsic component of Golgi membrane | 47 | 44 | 4.40 x 10^-02^ | 0.83 |
|  | Mitochondrial outer membrane translocase complex | 10 | 10 | 4.50 x 10^-02^ | 0.83 |
|  | Intrinsic component of mitochondrial outer membrane | 21 | 20 | 4.60 x 10^-02^ | 0.83 |
|  | Verylowdensity lipoprotein particle | 18 | 12 | 4.60 x 10^-02^ | 0.83 |
|  | Outer mitochondrial membrane protein complex | 14 | 14 | 4.60 x 10^-02^ | 0.83 |
|  | Nuclear chromosome, telomeric region | 116 | 99 | 4.90 x 10^-02^ | 0.84 |
|  | Pcg protein complex | 46 | 41 | 4.90 x 10^-02^ | 0.84 |
|  |  |  |  |  |  |
| ***Cellular components*** | |  |  |  |  |
|  | Kinase regulator activity | 206 | 181 | 1.00 x 10^-03^ | 0.78 |
|  | Kinase inhibitor activity | 95 | 86 | 1.70 x 10^-03^ | 0.78 |
|  | Sulfurtransferase activity | 12 | 11 | 2.30 x 10^-03^ | 0.78 |
|  | Protein tyrosine kinase activity | 176 | 149 | 3.70 x 10^-03^ | 0.78 |
|  | Chemorepellent activity | 21 | 18 | 4.20 x 10^-03^ | 0.78 |
|  | ADP binding | 31 | 30 | 5.20 x 10^-03^ | 0.78 |
|  | AMP binding | 9 | 9 | 5.60 x 10^-03^ | 0.78 |
|  | RNA polymerase binding | 53 | 51 | 6.00 x 10^-03^ | 0.78 |
|  | Trna methyltransferase activity | 26 | 26 | 6.60 x 10^-03^ | 0.78 |
|  | Protein tyrosine kinase activator activity | 16 | 16 | 8.60 x 10^-03^ | 0.91 |
|  | Mrna 5'UTR binding | 21 | 20 | 1.12 x 10^-02^ | 0.92 |
|  | Potassiumtransporting atpase activity | 11 | 10 | 1.20 x 10^-02^ | 0.92 |
|  | Receptor activator activity | 14 | 12 | 1.20 x 10^-02^ | 0.92 |
|  | Protein serine/threonine kinase inhibitor activity | 31 | 28 | 1.21 x 10^-02^ | 0.92 |
|  | Transferase activity, transferring alkyl or aryl (other than methyl) groups | 56 | 45 | 1.40 x 10^-02^ | 0.93 |
|  | RNA stemloop binding | 13 | 13 | 1.40 x 10^-02^ | 0.93 |
|  | Kinase activator activity | 75 | 74 | 1.70 x 10^-02^ | 1.00 |
|  | Sodium potassiumexchanging atpase activity | 11 | 10 | 2.30 x 10^-02^ | 1.00 |
|  | Quaternary ammonium group transmembrane transporter activity | 10 | 7 | 2.50 x 10^-02^ | 1.00 |
|  | Cyclindependent protein serine/threonine kinase regulator activity | 29 | 27 | 2.80 x 10^-02^ | 1.00 |
|  | Bhlh transcription factor binding | 26 | 25 | 2.80 x 10^-02^ | 1.00 |
|  | S100 protein binding | 14 | 13 | 2.90 x 10^-02^ | 1.00 |
|  | Intramolecular transferase activity | 25 | 24 | 3.40 x 10^-02^ | 1.00 |
|  | Poly(A) binding | 15 | 14 | 3.60 x 10^-02^ | 1.00 |
|  | Sulfuric ester hydrolase activity | 11 | 11 | 3.70 x 10^-02^ | 1.00 |
|  | Transcription corepressor activity | 207 | 189 | 4.00 x 10^-02^ | 1.00 |
|  | Voltagegated sodium channel activity | 23 | 16 | 4.60 x 10^-02^ | 1.00 |
|  | 3',5'cyclicamp phosphodiesterase activity | 15 | 15 | 4.80 x 10^-02^ | 1.00 |
|  | Alcohol binding | 78 | 71 | 4.80 x 10^-02^ | 1.00 |
|  | FK506 binding | 14 | 14 | 4.90 x 10^-02^ | 1.00 |
|  | Lipoprotein transporter activity | 12 | 10 | 4.90 x 10^-02^ | 1.00 |

^a^ Empirical p-value of the association test with DTC risk at the pathway level.

^b^ p-value of the association test with DTC risk at the pathway level, after FDR correction.

**Supplementary Table S5.** Similarity between associated pathways (*P*_EMP_<0.05) across databases.

| **KEGG pathway** | ***P*_EMP_^a^** | **Reactome pathway** | ***P*_EMP_^a^** | **KEGG Genes (*N*)** | **Reactome Genes (*N*)** | **Common genes (*N*)** | **Similarity score (Jaccard Index)** |
| --- | --- | --- | --- | --- | --- | --- | --- |
| Steroid biosynthesis | 1.30 x 10^-02^ | Cholesterol biosynthesis | 3.00 x 10^-02^ | 17 | 21 | 10 | 0.36 |
| EGFR tyrosine kinase inhibitor resistance | 3.70 x 10^-02^ | Signaling by ERBB2 | 7.80 x 10^-03^ | 77 | 46 | 18 | 0.17 |
| Cell cycle | 2.40 x 10^-02^ | Cell Cycle | 4.60 x 10^-02^ | 115 | 412 | 71 | 0.16 |
| Cell cycle | 2.40 x 10^-02^ | Cyclin A:Cdk2associated events at S phase entry | 2.80 x 10^-02^ | 115 | 80 | 25 | 0.15 |
| EGFR tyrosine kinase inhibitor resistance | 3.70 x 10^-02^ | SHC1 events in ERBB2 signaling | 4.20 x 10^-02^ | 77 | 20 | 12 | 0.14 |
| Cell cycle | 2.40 x 10^-02^ | Cyclin E associated events during G1/S transition | 2.30 x 10^-02^ | 115 | 78 | 23 | 0.14 |
| Fat digestion and absorption | 4.80 x 10^-02^ | Digestion of dietary lipid | 1.60 x 10^-02^ | 31 | 4 | 4 | 0.13 |
| Adipocytokine signaling pathway | 4.60 x 10^-02^ | mTOR signalling | 2.20 x 10^-02^ | 64 | 36 | 11 | 0.12 |
| EGFR tyrosine kinase inhibitor resistance | 3.70 x 10^-02^ | Signaling by VEGF | 1.60 x 10^-02^ | 77 | 96 | 19 | 0.12 |
| Cellular senescence | 1.40 x 10^-02^ | Cyclin E associated events during G1/S transition | 2.30 x 10^-02^ | 135 | 78 | 22 | 0.12 |
| Cellular senescence | 1.40 x 10^-02^ | Cyclin A:Cdk2associated events at S phase entry | 2.80 x 10^-02^ | 135 | 80 | 22 | 0.11 |
| Adipocytokine signaling pathway | 4.60 x 10^-02^ | Energy dependent regulation of mTOR by LKB1AMPK | 1.30 x 10^-02^ | 64 | 26 | 9 | 0.11 |
| EGFR tyrosine kinase inhibitor resistance | 3.70 x 10^-02^ | Intracellular signaling by second messengers | 2.60 x 10^-02^ | 77 | 236 | 31 | 0.11 |
| EGFR tyrosine kinase inhibitor resistance | 3.70 x 10^-02^ | GRB2 events in ERBB2 signaling | 3.10 x 10^-02^ | 77 | 14 | 9 | 0.11 |
| EGFR tyrosine kinase inhibitor resistance | 3.70 x 10^-02^ | PI3K events in ERBB2 signaling | 1.90 x 10^-02^ | 77 | 14 | 9 | 0.11 |
| MAPK signaling pathway | 3.10 x 10^-02^ | Intracellular signaling by second messengers | 2.60 x 10^-02^ | 222 | 236 | 45 | 0.11 |
| Adipocytokine signaling pathway | 4.60 x 10^-02^ | Activation of PPARGC1A (PGC1alpha) by phosphorylation | 1.20 x 10^-02^ | 64 | 9 | 7 | 0.11 |
| **KEGG pathway** | ***P*_EMP_^a^** | **GO biological process definition** | ***P*_EMP_^a^** | **KEGG Genes (*N*)** | **GO Genes (*N*)** | **Common genes (*N*)** | **Similarity score (Jaccard Index)** |
| One carbon pool by folate | 3.90 x 10^-02^ | tetrahydrofolate metabolic process | 2.20 x 10^-02^ | 18 | 18 | 14 | 0.64 |
| One carbon pool by folate | 3.90 x 10^-02^ | folic acidcontaining compound metabolic process | 5.00 x 10^-02^ | 18 | 25 | 14 | 0.48 |
| Transfer RNA biogenesis | 5.00 x 10^-02^ | tRNA processing | 4.30 x 10^-02^ | 138 | 110 | 80 | 0.48 |
| Amino sugar and nucleotide sugar metabolism | 1.30 x 10^-02^ | nucleotidesugar biosynthetic process | 3.30 x 10^-02^ | 45 | 22 | 21 | 0.46 |
| One carbon pool by folate | 3.90 x 10^-02^ | tetrahydrofolate interconversion | 6.30 x 10^-03^ | 18 | 9 | 8 | 0.42 |
| Transfer RNA biogenesis | 5.00 x 10^-02^ | tRNA modification | 2.40 x 10^-02^ | 138 | 71 | 50 | 0.31 |
| Transfer RNA biogenesis | 5.00 x 10^-02^ | RNA modification | 1.09 x 10^-02^ | 138 | 121 | 53 | 0.26 |
| Cholesterol metabolism | 5.90 x 10^-03^ | sterol transport | 3.10 x 10^-02^ | 43 | 77 | 24 | 0.25 |
| Glycine, serine and threonine metabolism | 4.80 x 10^-02^ | Lserine metabolic process | 4.40 x 10^-02^ | 31 | 10 | 8 | 0.24 |
| Glycine, serine and threonine metabolism | 4.80 x 10^-02^ | glycine metabolic process | 2.10 x 10^-02^ | 31 | 12 | 8 | 0.23 |
| MAPK signaling pathway | 3.10 x 10^-02^ | regulation of MAP kinase activity | 5.20 x 10^-03^ | 222 | 273 | 76 | 0.18 |
| Carbohydrate digestion and absorption | 4.70 x 10^-02^ | establishment or maintenance of transmembrane electrochemical gradient | 4.00 x 10^-02^ | 35 | 12 | 7 | 0.18 |
| Carbohydrate digestion and absorption | 4.70 x 10^-02^ | sodium ion export across plasma membrane | 1.90 x 10^-02^ | 35 | 12 | 7 | 0.18 |
| Glyoxylate and dicarboxylate metabolism | 2.50 x 10^-02^ | glycine metabolic process | 2.10 x 10^-02^ | 30 | 12 | 6 | 0.17 |
| Fat digestion and absorption | 4.80 x 10^-02^ | lipid digestion | 3.00 x 10^-02^ | 31 | 12 | 6 | 0.16 |
| Fat digestion and absorption | 4.80 x 10^-02^ | intestinal lipid absorption | 4.20 x 10^-02^ | 31 | 12 | 6 | 0.16 |
| MAPK signaling pathway | 3.10 x 10^-02^ | activation of protein kinase activity | 1.10 x 10^-02^ | 222 | 269 | 67 | 0.16 |
| One carbon pool by folate | 3.90 x 10^-02^ | glycine metabolic process | 2.10 x 10^-02^ | 18 | 12 | 4 | 0.15 |
| Transfer RNA biogenesis | 5.00x 10^-02^ | tRNA methylation | 1.20 x 10^-02^ | 138 | 30 | 22 | 0.15 |
| Carbohydrate digestion and absorption | 4.70 x 10^-02^ | export across plasma membrane | 4.60 x 10^-02^ | 35 | 20 | 7 | 0.15 |
| Steroid biosynthesis | 1.30 x 10^-02^ | sterol biosynthetic process | 1.30 x 10^-02^ | 17 | 63 | 10 | 0.14 |
| Transfer RNA biogenesis | 5.00 x 10^-02^ | RNA methylation | 3.80 x 10^-02^ | 138 | 56 | 23 | 0.13 |
| Adipocytokine signaling pathway | 4.60 x 10^-02^ | fatty acid transmembrane transport | 5.40 x 10^-04^ | 64 | 15 | 9 | 0.13 |
| Insulin resistance | 3.90 x 10^-02^ | regulation of fatty acid metabolic process | 1.80 x 10^-02^ | 94 | 73 | 19 | 0.13 |
| Cholesterol metabolism | 5.90x 10^-03^ | sterol metabolic process | 1.60 x 10^-02^ | 43 | 130 | 19 | 0.12 |
| Adipocytokine signaling pathway | 4.60 x 10^-02^ | regulation of fatty acid metabolic process | 1.80 x 10^-02^ | 64 | 73 | 15 | 0.12 |
| Cholesterol metabolism | 5.90x 10^-03^ | lipid digestion | 3.00 x 10^-02^ | 43 | 12 | 6 | 0.12 |
| Cholesterol metabolism | 5.90x 10^-03^ | intestinal lipid absorption | 4.20 x 10^-02^ | 43 | 12 | 6 | 0.12 |
| Adipocytokine signaling pathway | 4.60 x 10^-02^ | regulation of fatty acid oxidation | 2.70 x 10^-03^ | 64 | 28 | 10 | 0.12 |
| Adipocytokine signaling pathway | 4.60 x 10^-02^ | lipid oxidation | 2.10 x 10^-02^ | 64 | 93 | 17 | 0.12 |
| MAPK signaling pathway | 3.10 x 10^-02^ | activation of MAPK activity | 2.60 x 10^-02^ | 222 | 129 | 38 | 0.12 |
| Cell cycle | 2.40 x 10^-02^ | regulation of cyclindependent protein kinase activity | 2.60 x 10^-02^ | 115 | 79 | 21 | 0.12 |
| Fat digestion and absorption | 4.80 x 10^-02^ | longchain fatty acid transport | 2.80 x 10^-02^ | 31 | 55 | 9 | 0.12 |
| Carbohydrate digestion and absorption | 4.70 x 10^-02^ | potassium ion import | 2.20 x 10^-02^ | 35 | 33 | 7 | 0.11 |
| Adipocytokine signaling pathway | 4.60 x 10^-02^ | longchain fatty acid transport | 2.80 x 10^-02^ | 64 | 55 | 12 | 0.11 |
| Insulin resistance | 3.90 x 10^-02^ | longchain fatty acid transport | 2.80 x 10^-03^ | 94 | 55 | 15 | 0.11 |
| Adipocytokine signaling pathway | 4.60 x 10^-02^ | regulation of fatty acid betaoxidation | 1.32 x 10^-02^ | 64 | 16 | 8 | 0.11 |
| Adipocytokine signaling pathway | 4.60 x 10^-02^ | fatty acid transport | 1.50 x 10^-02^ | 64 | 78 | 14 | 0.11 |
| Cell cycle | 2.40 x 10^-02^ | cell cycle arrest | 2.10 x 10^-02^ | 115 | 207 | 31 | 0.11 |
| MAPK signaling pathway | 3.10 x 10^-02^ | peptidyltyrosine modification | 3.90 x 10^-02^ | 222 | 280 | 47 | 0.10 |
| Insulin resistance | 3.90 x 10^-02^ | fatty acid transport | 1.50 x 10^-02^ | 94 | 78 | 16 | 0.10 |
| Insulin resistance | 3.90 x 10^-02^ | regulation of lipid catabolic process | 4.90 x 10^-02^ | 94 | 46 | 13 | 0.10 |
| Fat digestion and absorption | 4.80 x 10^-02^ | fatty acid transport | 1.50 x 10^-02^ | 31 | 78 | 10 | 0.10 |
| Insulin resistance | 3.90 x 10^-02^ | fatty acid transmembrane transport | 5.40 x 10^-04^ | 94 | 15 | 10 | 0.10 |
| **KEGG pathway** | ***P*_EMP_^a^** | **GO cellular component definition** | ***P*_EMP_^a^** | **KEGG Genes (*N*)** | **GO Genes (*N*)** | **Common genes (*N*)** | **Similarity score (Jaccard Index)** |
| Cholesterol metabolism | 5.90 x 10^-03^ | proteinlipid complex | 2.20 x 10^-02^ | 43 | 31 | 13 | 0.21 |
| Carbohydrate digestion and absorption | 4.70 x 10^-02^ | sodium potassiumexchanging ATPase complex | 1.30 x 10^-02^ | 35 | 9 | 7 | 0.19 |
| Carbohydrate digestion and absorption | 4.70 x 10^-02^ | cationtransporting ATPase complex | 1.50 x 10^-02^ | 35 | 13 | 7 | 0.17 |
| Carbohydrate digestion and absorption | 4.70 x 10^-02^ | ATPase dependent transmembrane transport complex | 2.60 x 10^-02^ | 35 | 19 | 7 | 0.15 |
| Cholesterol metabolism | 5.90x 10^-03^ | verylowdensity lipoprotein particle | 4.60 x 10^-02^ | 43 | 12 | 6 | 0.12 |
| Cholesterol metabolism | 5.90x 10^-03^ | triglyceriderich plasma lipoprotein particle | 3.70 x 10^-02^ | 43 | 12 | 6 | 0.12 |
| Adipocytokine signaling pathway | 4.60 x 10^-02^ | nucleotideactivated protein kinase complex | 2.10 x 10^-03^ | 64 | 10 | 7 | 0.10 |
| **KEGG pathway** | ***P*_EMP_^a^** | **GO molecular function definition** | ***P*_EMP_^a^** | **KEGG Genes (*N*)** | **GO Genes (*N*)** | **Common genes (*N*)** | **Similarity score (Jaccard Index)** |
| Carbohydrate digestion and absorption | 4.70 x 10^-02^ | sodium potassiumexchanging ATPase activity | 2.30 x 10^-02^ | 35 | 10 | 7 | 0.18 |
| Carbohydrate digestion and absorption | 4.70 x 10^-02^ | potassiumtransporting ATPase activity | 1.20 x 10^-02^ | 35 | 10 | 7 | 0.18 |
| Glycosaminoglycan degradation | 4.30 x 10^-02^ | sulfuric ester hydrolase activity | 3.70 x 10^-02^ | 15 | 11 | 4 | 0.18 |
| Transfer RNA biogenesis | 5.00 x 10^-02^ | tRNA methyltransferase activity | 6.60 x 10^-03^ | 138 | 26 | 19 | 0.13 |
| Cholesterol metabolism | 5.90 x 10^-03^ | alcohol binding | 4.80 x 10^-02^ | 43 | 71 | 12 | 0.12 |
| MAPK signaling pathway | 3.10 x 10^-02^ | protein tyrosine kinase activity | 3.70 x 10^-03^ | 222 | 149 | 37 | 0.11 |

^a^ Empirical p-value of the association test with DTC risk at the pathway level.

Number of genes in pathway and number of common genes for each pair are shown.
